# Emerging changes in lake temperature extremes and variability in South America

**DOI:** 10.1007/s10584-026-04137-0

**Published:** 2026-02-16

**Authors:** Dieu Anh Dinh, Yan Tong, Lian Feng, Ayan Fleischmann, Eleanor Jennings, Valerie McCarthy, Siobhan Jordan, R. Iestyn Woolway

**Affiliations:** 1 Centre for Freshwater and Environmental Studies, Dundalk Institute of Technology, Dundalk, Ireland; 2 School of Environmental Science and Engineering, Southern University of Science and Technology, Shenzhen, China; 3 Mamirauá Institute for Sustainable Development, Tefé, Brazil; 4 School of History and Geography, Dublin City University, Dublin, Ireland; 5 School of Ocean Sciences, Bangor University, Menai Bridge, Anglesey, Wales

**Keywords:** Lake water temperature, Lake heatwave, Climate change, Climate projections, South american lakes

## Abstract

South America contains some of the world’s most ecologically and hydrologically diverse freshwater systems, which are increasingly vulnerable to climate change and human pressures. Despite their importance, the diurnal and interannual variability of lake surface water temperature (LSWT) across the continent remains poorly understood. In this study, we analyze thermal patterns in 2,406 South American lakes, spanning both historical (1981–2020) and future (2021–2099) periods. We assess LSWT trends, lake heatwave dynamics, and the influence of key meteorological drivers on lake thermal dynamics. Our results show that 97.0% of lakes (*n* = 2,333) experienced significant warming over the past four decades (+ 0.11 K decade^− 1^), with 86.2% (*n* = 2,074) also exhibiting rising diurnal temperature variability (+ 0.02 K decade^− 1^). Air temperature was the dominant driver in the northern and southern regions, while shortwave radiation played a greater role in shaping diurnal dynamics. LSWT anomalies are projected to rise, with heatwave events increasing in frequency, duration and intensity by the end of the 21st century. Additionally, we introduce a novel thermal typology based on diurnal and seasonal temperature ranges, identifying distinct lake response types, such as Thermally Extreme and Thermally Buffered, highlighting differential sensitivities to climate forcing. These findings provide new insight into lake thermal behaviour under climate change and underscore the need for targeted adaptation and conservation strategies to protect freshwater ecosystems in South America.

**Supplementary Information:**

The online version contains supplementary material available at 10.1007/s10584-026-04137-0.

## Introduction

Lakes are widely recognized as sentinels of climate change, responding rapidly to atmospheric and hydrological shifts (Adrian et al. 2009). Changes in lake surface water temperature (LSWT), ice cover, and water levels serve as notable early warning indicators of broader climatic transformations (Castendyk et al. 2016; Vinnå et al. 2021; Zhang and Duan 2021). Among these indicators, LSWT is particularly critical due to its role in regulating heat balance and moisture exchange between lakes and their surroundings (Schmid and Read 2022). It also influences essential physical, chemical, and biological processes, including thermal stratification, oxygen dynamics, and ecosystem productivity (Schmid et al. 2014; Jane et al. 2021; Kraemer et al. 2021). Therefore, understanding long-term LSWT variations is essential for assessing how lake ecosystems respond to climate change.

Global LSWT trends are primarily driven by rising air temperatures, with consistent evidence of warming across diverse geographical regions (Schneider and Hook 2010; O’Reilly et al. 2015; Tong et al. 2023; Piccolroaz et al. 2024). However, LSWT patterns exhibit considerable regional variability, influenced by climatic drivers such as solar radiation (Schmid and Köster 2016) and wind speed (Woolway et al. 2019), as well as intrinsic lake characteristics like surface area and depth (Kraemer et al. 2015; Augusto-Silva et al. 2019; Zhou et al. 2024). In addition to long-term warming, extreme events such as lake heatwaves, defined as prolonged periods of anomalously high surface water temperatures, are becoming more frequent (Woolway et al. 2021a, 2022; Wang et al. 2023, 2024). These extreme events can intensify thermal stratification (Woolway et al. 2020), exacerbate hypoxia in deeper layers, and disrupt metabolic and microbial processes, thereby altering nutrient cycling and oxygen dynamics (Jankowski et al. 2006; North et al. 2014). Such disruptions may trigger harmful algal blooms and mass mortality events among aquatic species (Ho et al. 2019; Till et al. 2019; Mendes et al. 2024; Fleischmann et al. 2024). With ongoing global warming, lake heatwaves are projected to increase in frequency, duration, and intensity throughout the 21^st^ century (Woolway et al. 2021a; Wang et al. 2023; Zhang and Yao 2023).

South America hosts some of the world’s most diverse and expansive freshwater systems, including the Amazon River, the largest river system by discharge, and numerous high-altitude lakes along the Andes, the longest continental mountain range (Buytaert and Breuer 2013). Lakes, rivers, and wetlands are integral to the continent’s hydrological cycle, serving as critical water sources for ecosystems, agriculture, and human populations (Hamilton et al. 2002; Junk 2013; Kandus et al. 2018; Siqueira et al. 2018; Fleischmann et al. 2021). However, South American freshwater resources are increasingly threatened by climate change and human activities (Buytaert and Breuer 2013; Junk 2013). Over recent decades, South America has experienced significant increases in air temperature (World Meteorological Organization 2021), a trend projected to continue and likely to cause profound impacts on LSWT (Margin et al. 2014; Llopart et al. 2020). Despite the continent’s ecological importance and vulnerability to climate change, research on LSWT dynamics and lake heatwaves across South America remains limited. Most studies have focused on specific water bodies, particularly glacial lakes (Quade and Kaplan 2017; Wilson et al. 2018; Mergili et al. 2020; Hata et al. 2022) or on changes in lake water level (Zolá and Bengtsson 2006; Pasquini et al. 2008; Carabajal and Boy 2021). The relative scarcity of studies addressing LSWT trends and lake heatwave dynamics on a regional scale is partly due to limited in situ measurements, particularly in the Southern Hemisphere (Aranda et al. 2021). This results in major knowledge gaps in understanding how LSWT and thermal extremes are evolving across different regions and lake types.

To address these gaps, this study investigates historical (1981–2020) and projected future (2021–2099) changes in LSWT and lake heatwave patterns across South America using satellite-derived and modelled data (Tong et al. 2023). We examine LSWT trends, lake heatwave characteristics (duration, intensity, and cumulative intensity), and the role of meteorological drivers including air temperature, solar and longwave radiation, wind speed, and humidity. In addition to long-term trends, this study explores short- and medium-term thermal variability by introducing a novel typology of lake responses based on diurnal and seasonal temperature range (DTR and STR, respectively). DTR captures short-term fluctuations within a 24-hour cycle, while STR reflects annual-scale thermal variability. Together, they provide complementary insights into how lakes respond to atmospheric forcing. By classifying lakes according to combinations of DTR and STR, we identify distinct thermal response types, offering a new framework for assessing lake sensitivity to climate change across multiple timescales.

## Study area

South America encompasses a vast and diverse range of lake systems spread across its broad latitudinal and longitudinal extent (12°28’N–55°59’S; 30°W–80°W) (Nuñez-Hidalgo et al. 2023). These lakes span various climatic and geographic regions, from tropical lowlands and extensive floodplain systems in the Amazon Basin to temperate zones and high-altitude glacial environments along the Andes, the longest continental mountain range on Earth (Siqueira et al. 2018; Carabajal and Boy 2021). This climatic diversity influences not only the thermal dynamics of lakes but also their hydrological regimes and ecological characteristics. The continent’s lakes exhibit considerable variability in size, volume, and elevation. Surface areas range from small ponds measuring approximately 0.1 km^2^ in surface areas to large water bodies such as Lake Titicaca, which spans 8,003 km^2^, while lake volumes vary between 0.0001 km^3^ and 893 km^3^ (Messager et al. 2016). Prominent systems include Amazon floodplain lakes, which experience dramatic seasonal water level fluctuations, and Andean glacial lakes that are particularly sensitive to temperature changes and glacial melt.

Lakes across South America play critical roles in the hydrological cycle, serving as essential freshwater resources for agriculture, industry, hydropower generation, and drinking water supply. They also support rich biodiversity, including endemic and economically significant species, while holding cultural and societal importance for numerous communities (Llames and Zagarese 2009). However, these freshwater systems are increasingly threatened by climate change, pollution, and anthropogenic activities such as deforestation, mining, and agricultural expansion (Llames and Zagarese 2009). The diverse geographic distribution of lakes across South America offers a unique opportunity to investigate how LSWT responds to environmental changes across different climatic and altitudinal gradients. Understanding these patterns is essential for predicting future changes and developing effective adaptation strategies.

## Data and methodology

### GLAST dataset

The GLAST dataset provides LSWT data for 92,245 lakes worldwide from 1981 to 2020 (Tong et al. 2023). It was generated using a combination of satellite earth observation data and numerical modeling in four key steps: (i) Lake Selection: Lakes were identified based on permanent water bodies in the HydroLAKES database (Messager et al. 2016); (ii) Temperature Retrieval: LSWT was derived from Landsat satellite data using a statistical mono-window algorithm. With a spatial resolution of 60–120 m, temperatures were measured at least three pixels away from the shoreline to ensure accuracy; (iii) Model Calibration: The Freshwater Lake (FLake) model (Mironov et al. 2010) was calibrated using the Landsat-derived LSWT data (1981–2020). The model was fine-tuned for hourly temperature predictions using meteorological variables - including air temperature, wind speed, longwave and shortwave downward radiation, and specific humidity - obtained from ERA5-Land reanalysis dataset (Hersbach et al. 2020) at a grid resolution of 0.1° × 0.1°; (iv) Simulation and Validation: the calibrated FLake model was used to simulate hourly LSWT for each lake, with validation against in situ measurements resulting in a median absolute error (MAE) of 1.16 °C (Tong et al. 2023). In this study, we performed additional validation using field observations from two representative lakes: Lake Mascardi (Argentina) and Lake Tefé (Brazil) with MAE of 1.29 °C and 2.61 °C, respectively (Fig. S1). The comparison between FLake simulations and these in-situ observations reveals a general underestimation by the GLAST dataset for these specific South American lakes. However, it should be noted that the GLAST simulation represents a spatially aggregated whole-lake average, whereas the in-situ data are derived from single-point measurements from these meandering lakes. Using the hourly simulations, we estimated the DTR in the studied lakes, calculated as the difference between daytime and nighttime LSWT, as well as the seasonal and inter-annual variations in LSWT.

Future LSWT projections (2021–2099) were generated using an optimized lake-specific FLake model for each lake (i.e., relative to the satellite-derived LSWTs), forced by four global climate models (GCM, including IPSL-CM5A-LR, GFDL-ESM2M, MIROC5, and HadGEM2-ES) under three greenhouse gas emission scenarios: RCP 2.6 (low), RCP 6.0 (intermediate), and RCP 8.5 (high) (Tong et al. 2023). FLake simulations were conducted for each GCM, and the mean and standard deviation were estimated. In this study, LSWT anomalies were computed relative to the 1981–2006 baseline. Future LSWTs were also bias-corrected using a Quantile Delta Mapping (QDM) approach, which has been demonstrated as an effective method in a previous study on Lake Titicaca, South America (Dinh et al. 2026). As the GCM input data were only available at daily intervals, our future projections do not resolve diurnal temperature ranges.

### Lake heatwaves

The GLAST dataset was used to calculate lake heatwaves, defined as periods in which surface water temperatures increase higher than the seasonal 90th percentile for at least five consecutive days (Woolway et al. 2021b). Lake heatwave frequency was calculated during the ice-free season using the package “*heatwaveR*” in R (Schlegel and Smit 2018). Lake heatwave data were generated using three steps: (1) annual climatological mean and 90th percentile temperature threshold were calculated over the period of 1991–2020. An 11-day window centred on each date was used, followed by smoothing with a 31-day moving average; (2) Lake heatwaves were then calculated as defined above. If two events were separated by < 2 days, they were combined into a single event. If heatwaves extended to 2 or more calendar years, they were split on December 31, with a maximum length of 366 days per event; (3) We focused on four heatwave metrics including the total number of heatwave days (days), duration (days), intensity (the anomalies above the climatological mean; K) and cumulative intensity (K x days). These metrics were then aggregated annually to estimate heatwave trends.

### Lake classification

**Fig. 1:**
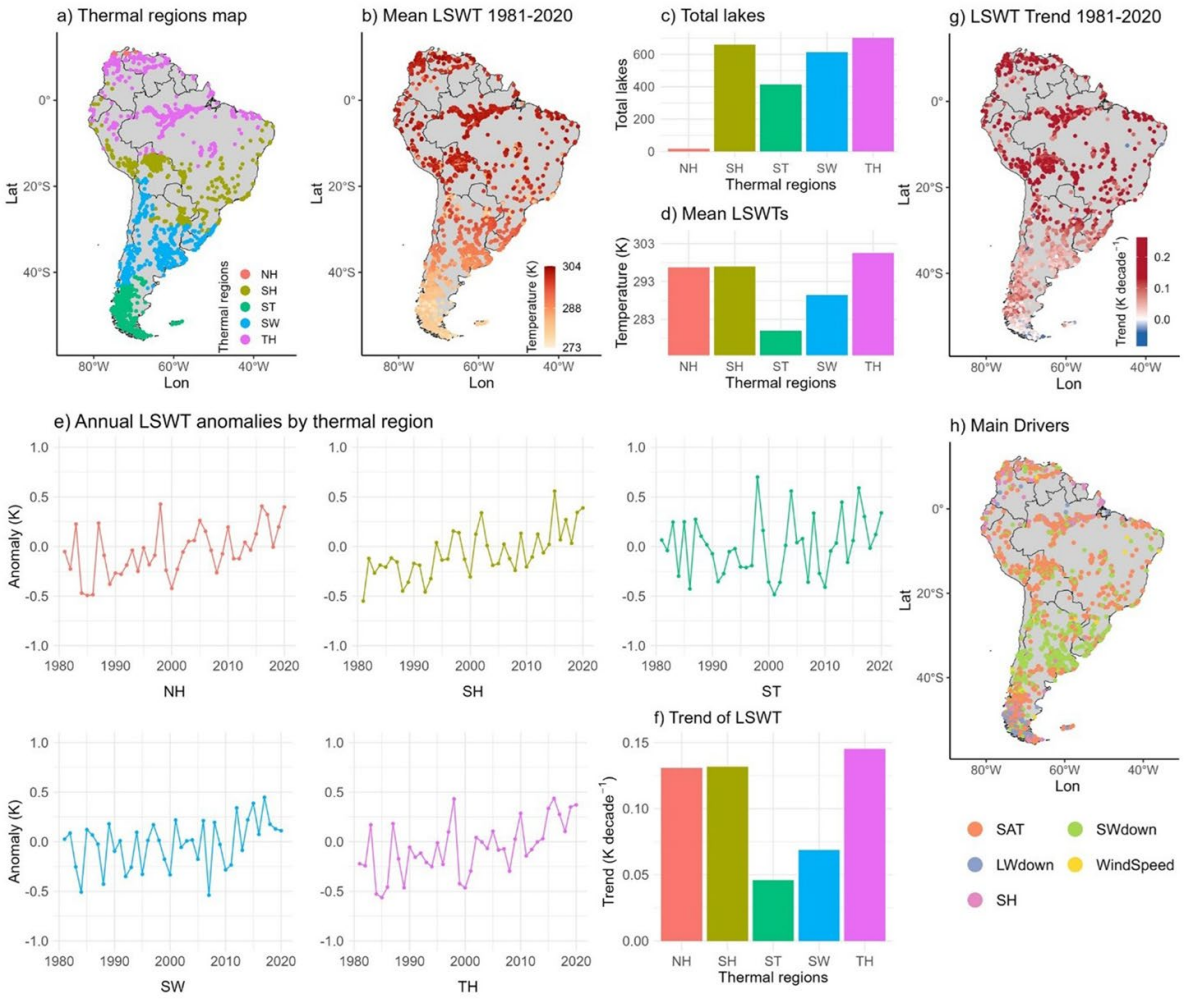
Temporal and spatial pattern of LSWTs during the historical period (1981-2020). (**a**) Maps of thermal regions in South America (NH- Northern Hot, SH- Southern Hot, ST- Southern Temperate, SW- Southern Warm, TH- Tropical Hot); (**b**) Average LSWT of each lake map; (**c**) Total number of lakes per thermal region; (**d**) Average LSWT per thermal region; (**e**) Annual LSWT anomaly per thermal region; (**f**) LSWT Trends per thermal region; (**g**) LSWT trend per lake; (**h**) Contribution of meteorological variables to LSWT trend map (SAT- Surface Air Temperature, SH- Specific Humidity, WindSpeed- Wind Speed, LWdown- Longwave downward radiation, SWdown- Shortwave downward radiation)

Lake data from GLAST was classified into different thermal regions following the methods developed by Maberly et al. (2020). This approach used satellite observations of LSWT from 732 lakes globally between 1996 and 2011 to categorise distinct lake regions. Firstly, mean LSWT of individual lakes were calculated. Secondly, a saturated b-spline based statistical modelling (de Boor 1980) was used to smooth out the LSWT of each lake over time. If ice was detected, the temperature under ice cover was set to 0 °C and the smooth function was adjusted accordingly. Thirdly, K-means clustering was used to group the lakes based on their temperature temporal patterns (seasonal patterns, mean temperature and long-term changes). Finally, nine thermal regions were classified using gap statistics (Tibshirani et al. 2002). These lake groups were also compared with other classification schemes (global climate and terrestrial ecoregions). Lakes were divided into 11 groups using the Koppen-Geiger climate classification (Peel et al. 2007). For the terrestrial ecoregions, lakes were classified into 13 groups (Olson et al. 2001). Furthermore, lake groups were compared with the air temperature clusters derived from bi-monthly temperature for the same number of LSWT from 1995 to 2012 (Harris et al. 2014). The air temperature clusters matched the LSWT clusters, grouped into nine classifications that corresponded as nearly as possible to lake thermal regions (Maberly et al. 2020). Following this approach, 2,406 lakes from the GLAST dataset were categorised into five lake thermal regions (Northern Hot [*n* = 17], Tropical Hot [*n* = 660], Southern Hot [*n* = 414], Southern Warm [*n* = 613] and Southern Temperate [*n* = 702]) (Fig. 1a and c).

### Diurnal to seasonal lake temperature typology

In this study, we developed a typology of lake thermal responses by classifying lakes according to their diurnal and seasonal temperature range (i.e., DTR and STR, respectively). The DTR was computed as the average difference between daytime and nighttime surface water temperatures across the observation period, capturing short-term thermal variability within a 24-hour cycle. In contrast, STR was defined as the difference between the annual maximum and minimum surface water temperatures, reflecting long-term seasonal thermal variability. To categorize lakes based on these metrics, we used percentile-based thresholds derived from the distribution of DTR, STR, and the DTR/STR ratio across all lakes in South America included in our dataset. Specifically, values below the 25th percentile were considered low, while those above the 75th percentile were considered high. These thresholds allowed us to systematically identify distinct thermal response patterns among lakes. Based on combinations of high and low values of DTR and STR, as well as their ratio, we defined four thermal typologies of lakes: (i) Diurnally Volatile Lakes (High DTR, Low STR): strong daily fluctuations but relatively stable across seasons; (ii) Diurnally Stable, Seasonally Volatile Lakes (Low DTR, High STR): muted daily variability but pronounced seasonal changes; (iii) Thermally Extreme Lakes (High DTR, High STR): large variations at both timescales; (iv) Thermally Buffered Lakes (Low DTR, Low STR): stable at both daily and seasonal scales. Lakes with both DTR and STR between the 25th and 75th percentiles were classified as “Others”, representing moderate variability that did not fit into the extreme categories. This framework provides insights into lake responses to thermal forcing on multiple timescales, facilitating comparative analyses and ecological interpretation.

### Assessing the impacts of meteorological drivers

To quantify the relative contributions of meteorological drivers—specifically surface air temperature, longwave and shortwave downward radiation, specific humidity, and wind speed—to the trends in LSWT and DTR, we employed the FLake model used in generating the GLAST dataset. As a process-based physical model, FLake simulates lake thermal dynamics by solving the energy budget equations of the water column based on atmospheric forcing and lake morphometry. This physical framework allows for the attribution of temperature trends to specific drivers through controlled sensitivity experiments. We conducted a total of six simulations for each lake to isolate driver contributions. First, a reference simulation was performed where the model was driven by the original historical meteorological forcing (1981–2020), preserving the observed long-term trends in all variables. Subsequently, five separate sensitivity simulations were conducted to isolate the impact of individual drivers. In each of these simulations, the original trend of one target variable was preserved, while the trends of all other meteorological variables were removed. Following methods established in previous attribution studies (Li et al. 2019; Tong et al. 2023), the detrending of non-target variables was achieved by repeating the meteorological forcing from the baseline year (1981) for the subsequent 40 years. The resulting trends in LSWT and DTR for each simulation were calculated using the Sen’s slope estimator and the Mann-Kendall significance test via the *trend* package (Pohlert 2023) in R (version 4.4) (R Core Team 2025). The relative contribution of each driver was then assessed by comparing the magnitude of the trends derived from the sensitivity simulations against the reference simulation.

## Results

### Historic patterns of change in LSWT, lake heatwaves and diurnal-seasonal variability

Between 1981 and 2020, mean annual LSWTs across South American lakes ranged from 273 K to 304 K, exhibiting substantial spatial variability (Fig. 1b). Warmer lakes were concentrated in the Tropical Hot (TH), Northern Hot (NH), and Southern Hot (SH) regions, while cooler lakes predominated in the Southern Temperate (ST) and Southern Warm (SW) zones (Fig. 1b and d). The TH region recorded the highest average LSWT at approximately 301 K. During this period, LSWT anomalies generally fluctuated between –1 K and +1 K, with notable deviations in the TH and SH regions (Fig. 1e). A warming trend was observed in 97.0% of the lakes (*n* = 2,333), averaging +0.11 K decade^-1^ (Fig. 1g), whereas only 3.0% (*n* = 73) exhibited marginal cooling at a rate of –0.02 K decade^-1^. Regionally, TH warmed the fastest (+0.15 K decade^-1^), followed by NH and SH (each +0.13 K decade^-1^; *p* < 0.05). Warming in ST and SW was lower, at +0.05 K decade^-1^ (not significant) and +0.07 K decade^-1^ (*p* < 0.05), respectively (Fig. 1f). Air temperature was identified as the primary driver of LSWT trends in most lakes (52.7%; *n* = 1,269). In contrast, shortwave downward radiation was the dominant driver in the SW region, affecting 30.8% of lakes (*n* = 741) (Fig. 1h; Fig. S2–S4). Other contributing variables included longwave radiation (7.2%), specific humidity (7.6%), and wind speed (1.6%).

**Fig. 2:**
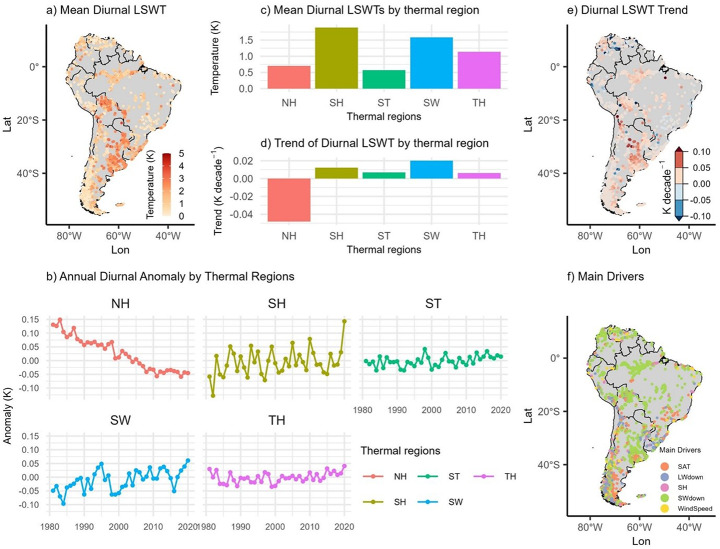
Temporal and spatial pattern of diurnal LSWTs during the historical period (1981-2020). (**a**) Average diurnal LSWT of each lake map; (**b**) Annual diurnal LSWT anomaly per thermal region; (**c**) Average diurnal LSWT per thermal region; (**d**) Diurnal LSWT Trends per thermal region; (**e**) Diurnal LSWT Trends map; (**f**) Contribution of meteorological variables to diurnal LSWT trend map

The DTR varied from ~0 to 5 K across lakes during the study period (Fig. 2a). The SH region exhibited the highest average DTR (1.9 K), followed by SW (1.6 K), TH (1.1 K), NH (0.7 K), and ST (0.6 K) (Fig. 2c). Most lakes (86.2%, *n* = 2,074) experienced increasing DTR trends, with a mean rate of +0.02 K decade^-1^, while the remainder (13.8%, *n* = 332) showed a modest decline of –0.03 K decade^-1^ (Fig. 2e). Although overall changes were small, regional trends were considerable. NH exhibited a notable decline (–0.05 K decade^-1^; *p* < 0.05), whereas other regions showed slight positive trends ranging from +0.01 K to +0.02 K decade^-1^ (Fig. 2b and d). Shortwave downward radiation was the dominant driver of DTR variability, influencing 66.8% of the lakes (*n* = 1,530) (Fig. 2f; Fig. S5–S7). Longwave radiation (13.4%) and air temperature (12.5%) also played important roles, with wind speed (5.8%) and specific humidity (4.7%) contributing to a lesser extent.

**Fig. 3:**
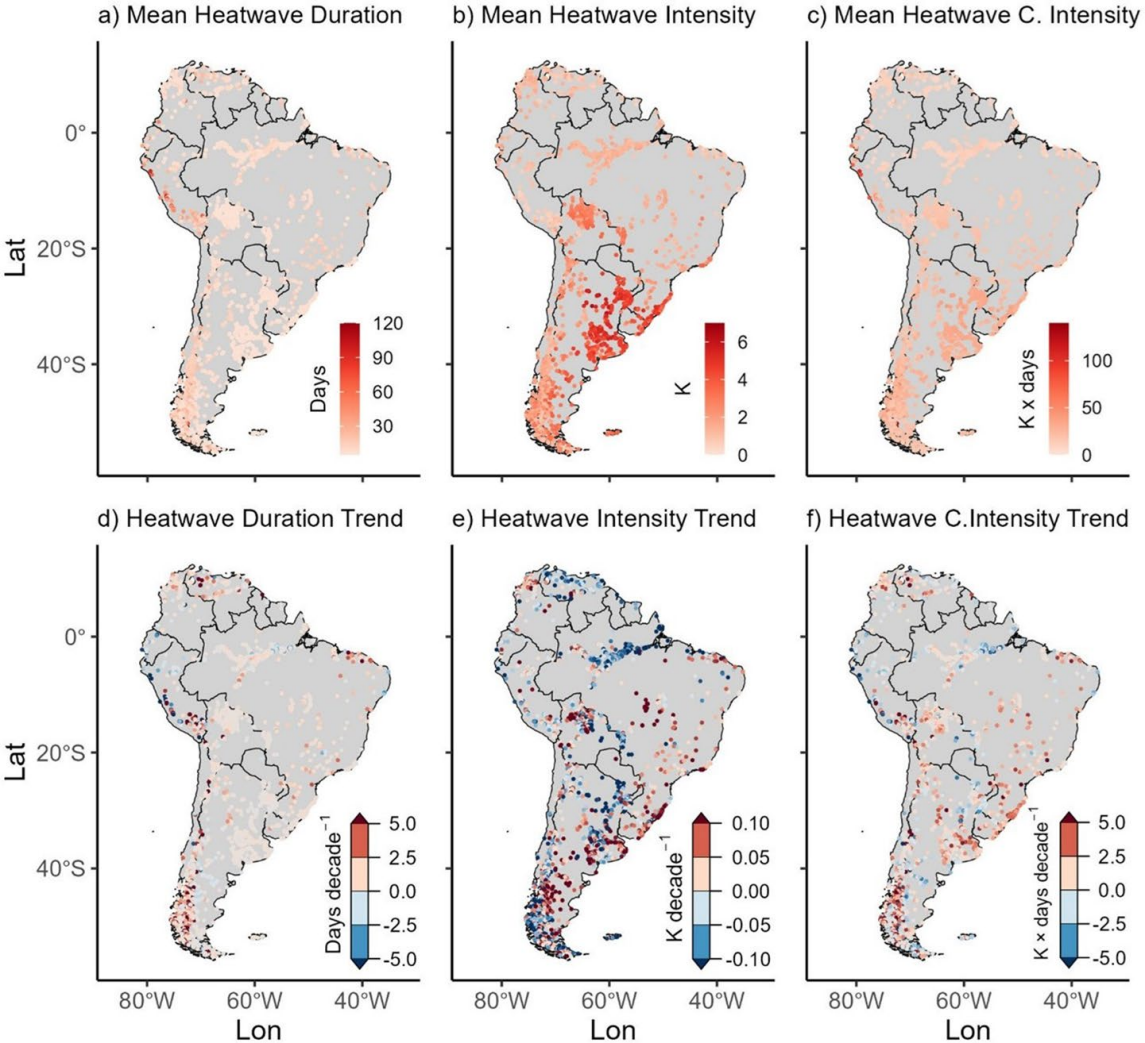
Historical mean heatwave (HW) and trend maps (1981-2020); (**a**) Mean HW duration, (**b**) Mean HW Intensity, (**c**) Mean HW Cumulative Intensity, (**d**) HW Duration Trend, (**e**) HW Intensity Trend and (**f**) HW Cumulative Intensity Trend

**Fig. 4:**
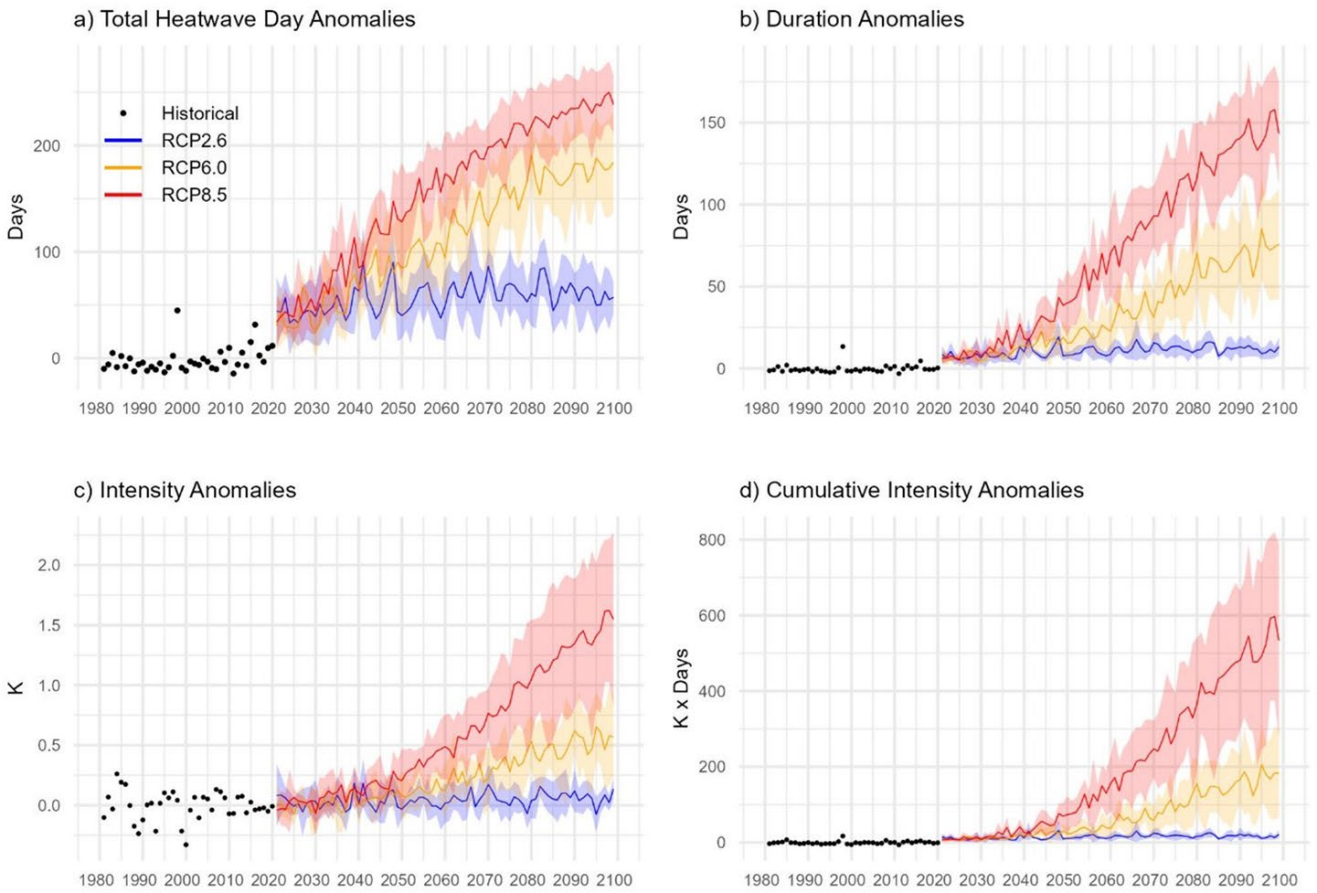
Historical and future projections of lake heatwave anomalies in the studied lakes under different RCPs, including (**a**) Total heatwave days, (**b**) Heatwave duration, (**c**) Heatwave intensity, and (**d**) Heatwave cumulative intensity. The black points represent anomaly values during the historical period from 1981 to 2020. The thick lines represent heatwave anomalies and the shaded areas show the standard deviation across the multi-model ensemble. The projections under RCP 2.6 (blue), RCP 6.0 (orange), and RCP 8.5 (red) scenarios from 2021 to 2099

Historic lake heatwaves ranged in duration from 5 to 116 days, with average intensities between 0.4 K and 6.3 K and cumulative intensities from 6 to 131 K x days (Fig. 3a–c). The SH and SW regions exhibited the highest heatwave intensities. Trends in lake heatwave characteristics were pronounced. A majority of lakes (79.5%, *n* = 1,912) showed increasing duration trends, and nearly half (47.8%, *n* = 1,151) experienced rising intensity trends at approximately +0.1 K decade^-1^ (Fig. 3d–f). Cumulative heatwave intensity increased in 63.0% of lakes (*n* = 1,516). Across the continent, the number of heatwave days showed a significant positive trend (+0.3 days decade^-1^), while changes in intensity were minor and not statistically significant (–3.7 × 10^-4^ K decade^-1^) (Fig. 4a-d).

**Fig. 5:**
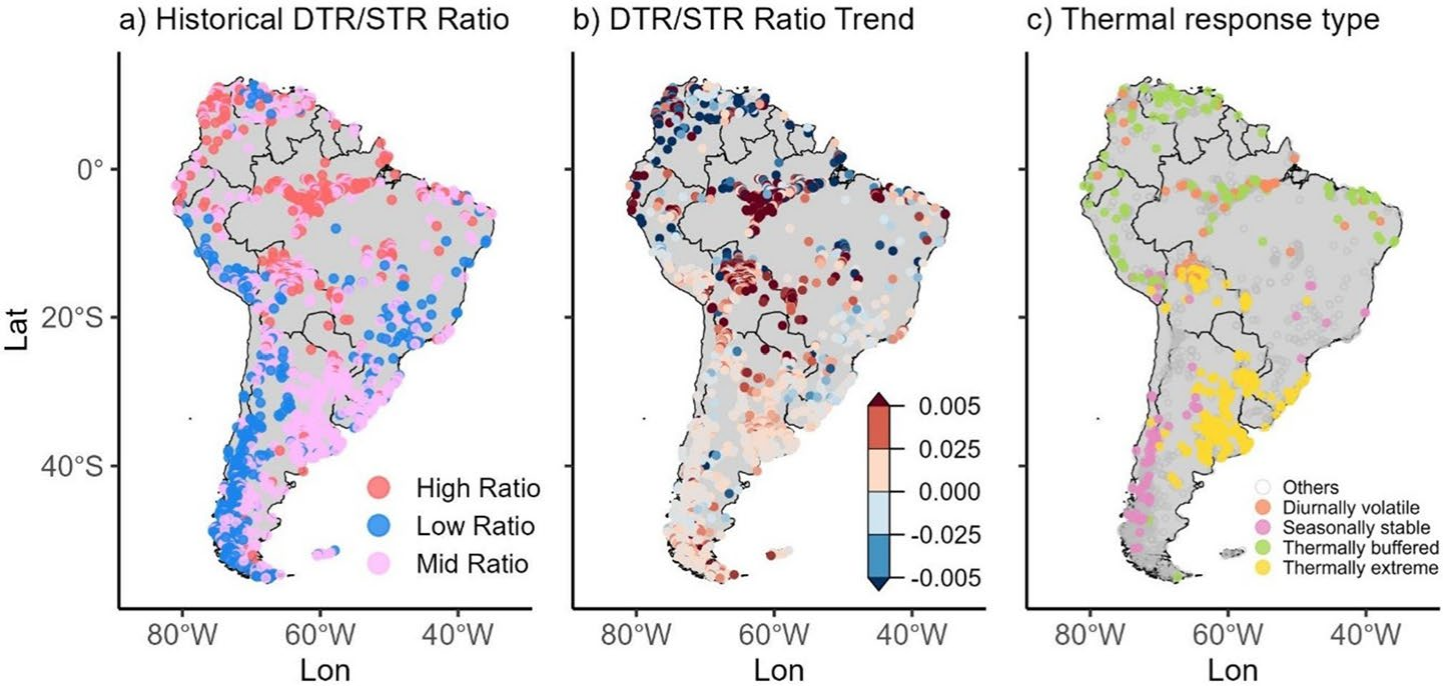
Diurnal-seasonal variability during the historical period (1981-2020): (**a**) Historical DTR/STR ratio; (**b**) DTR/STR trend per decade, and (**c**) Thermal response type (grey circle defined as “Others” type)

Lakes were further categorized in this study based on the ratio of DTR to STR, revealing distinct spatial patterns. Approximately 50% of lakes (*n* = 1,202) exhibited a mid-range DTR/STR ratio, while high and low ratio lakes each accounted for 25% (*n* = 602) (Fig. 5a). High-ratio lakes were concentrated in the Amazon and northern South America, mid-range lakes in the southeast, and low-ratio lakes in the Andes. Although DTR/STR trends ranged from –0.1 to +0.3 decade^-1^, most values clustered around ±0.005 decade^-1^ (Fig. 5b). Lakes in the north and east generally showed decreasing trends, while central South America exhibited the most prominent increases (>0.005 decade^-1^). Using DTR, STR, and their ratio, lakes were classified into four thermal response types: Thermally Extreme (*n* = 258), Thermally Buffered (*n* = 113), Seasonally Stable (*n* = 69), and Diurnally Volatile (*n* = 52), with the remainder (*n* = 1,914) categorized as “Others” (Fig. 5c). Thermally Extreme lakes were mostly in southeastern South America, Diurnally Volatile and Thermally Buffered lakes occurred mainly in the north (~12°N–20°S), and Seasonally Stable lakes were found primarily in the Patagonian Andes.

### Future changes during the 21st century

**Fig. 6:**
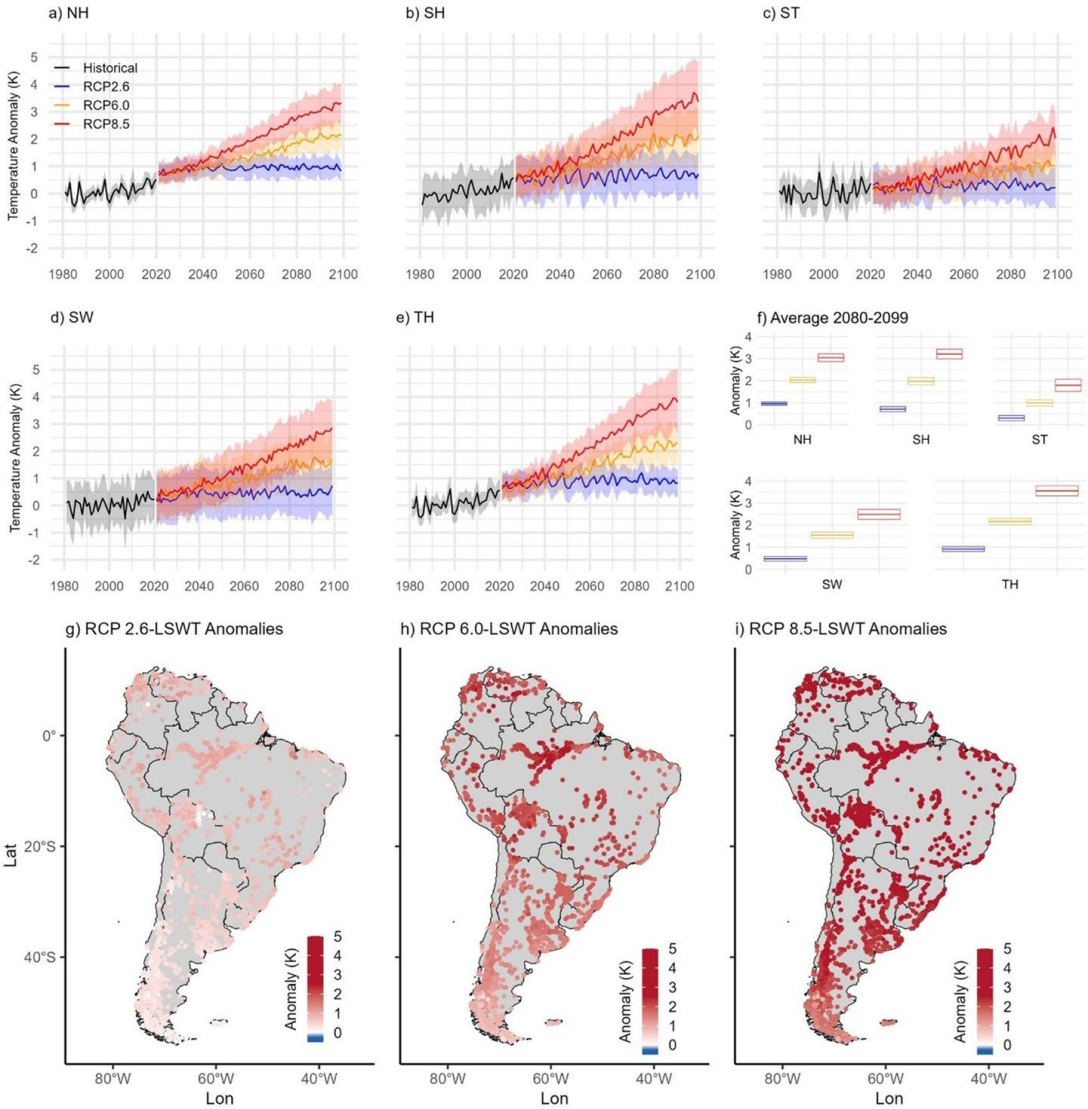
Historical and future projections of LSWT anomalies under different RCPs per thermal region: (**a**) NH, (**b**) SH, (**c**) ST, (**d**) SW and (**e**) TH. The thick lines represent mean temperature anomalies and the shaded areas show the standard deviation across the multi-model ensemble. The historical temperature anomalies (black) from 1981 to 2020, the projections under RCP 2.6 (blue), RCP 6.0 (orange), RCP 8.5 (red) scenarios from 2021 to 2099. (f) Average LSWT during the period of 2080-2099 per thermal region. (g-i) LSWT anomalies from 2080 to 2099 under RCP 2.6, RCP 6.0 and RCP 8.5 respectively

By the late 21^st^ century (2080–2099), LSWTs across South America are projected to increase substantially under all emission scenarios, with pronounced regional differences (Fig. 6a-f). Under the low-emission scenario (RCP 2.6), LSWTs are projected to increase by between 0.3 ± 0.1 K and 1.0 ± 0.1 K across the lake thermal regions. LSWT warming is projected to intensify further under the moderate-emission scenario (RCP 6.0), with projected increases ranging from 1.0 ± 0.1 K to 2.2 ± 0.1 K by 2080-2099 across thermal regions. Projections under the high-emission scenario (RCP 8.5) suggest the greatest warming, with temperature increases ranging from 1.8 ± 0.3 K in the ST region to 3.6 ± 0.2 K in the TH region. Overall, the TH region is projected to experience the greatest temperature increases across all scenarios, while the ST region consistently exhibits the lowest warming rates.

Future projections under various emission scenarios suggest substantial increases in heatwave intensity, frequency, and duration (Fig. 4a-d; Fig. S8-S11). Under RCP 2.6, relative to the 1981–2006 baseline, average heatwave intensity is projected to increase by 0.04 ± 0.06 K, with an increase in average duration of 11 ± 3 days, a total increase in heatwave days of 57 ± 14 days and a cumulative intensity of 15 ± 6 K x days. Under RCP 6.0, heatwave intensity is projected to increase by 0.2 ± 0.2 K, with a cumulative intensity increase of 69 ± 59 K x days. Additionally, the total number of heatwave days is projected to increase by 114 ± 51 days, and the average duration is expected to extend by 34 ± 24 days. The projections under RCP 8.5 suggest the most drastic changes. Under this scenario, heatwave intensity is anticipated to increase by 0.6 ± 0.5 K, average duration by 71 ± 50 days, total heatwave days by 157 ± 67 days and a cumulative intensity of 205 ± 185 K x days by the end of this century.

**Fig. 7:**
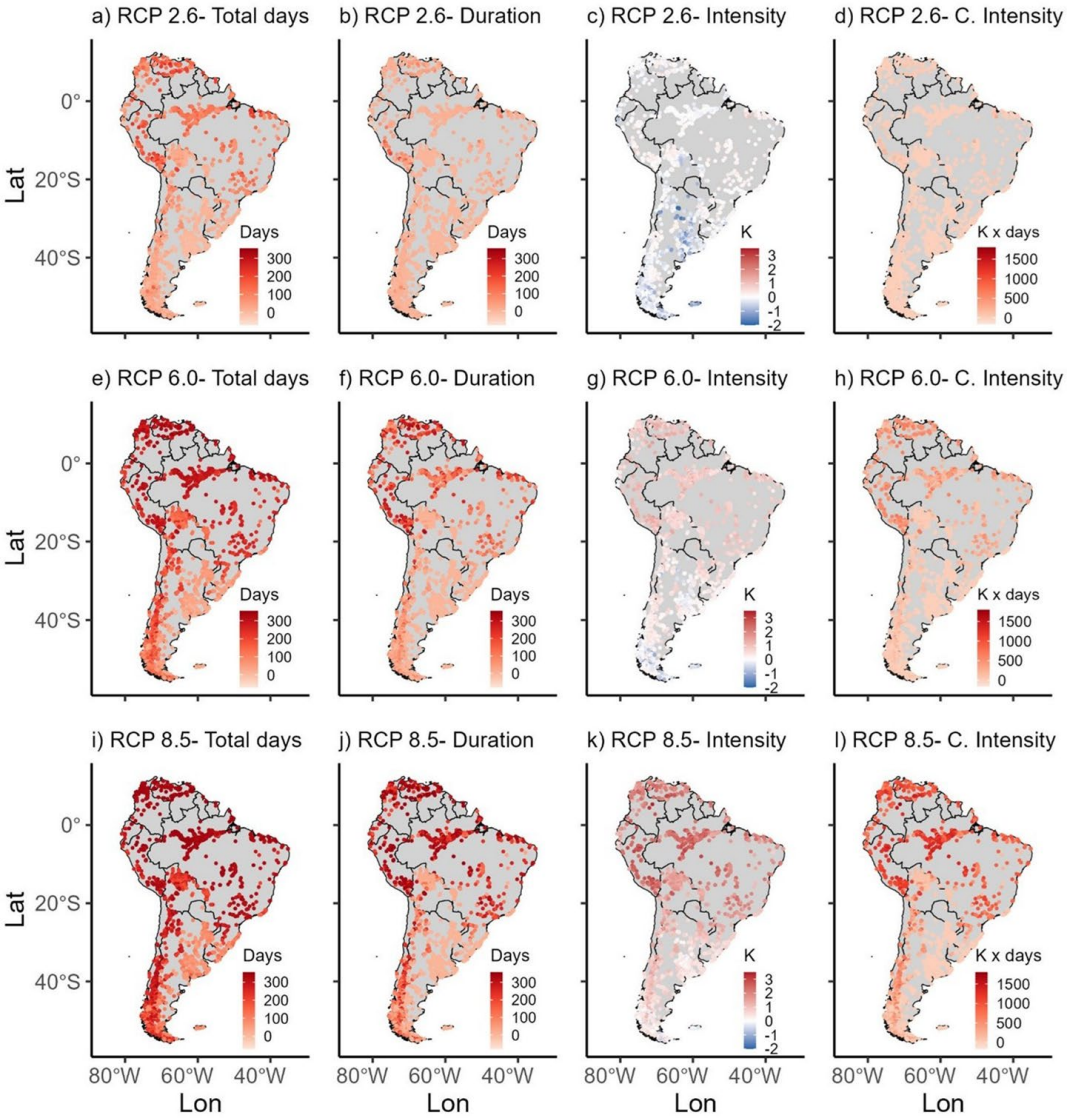
Future projections of lake heatwave anomalies (from left to right: total heatwave days, heatwave duration, heatwave intensity and cumulative intensity) of each studied lake under (**a-d**) RCP 2.6; (**e-h**) RCP 6.0; (**i-l**) RCP 8.5 scenarios between 2080 and 2099 relative to the 1981–2006 baseline

Spatial variability across the continent is notable, with northern regions, including the Amazon, and areas along the western Andes, experiencing the most substantial increases in heatwave frequency and intensity (Fig. 7a-l). Conversely, the southeastern region consistently exhibits smaller changes and, in some cases, even negative trends under some future scenarios. For example, under RCP 2.6, the total number of heatwave days ranges from a maximum decrease of 69 days to a maximum increase of 276 days by 2080-2099. Moreover, changes in heatwave duration vary from a decrease of 79 days to an increase of 214 days, while intensity changes range from a decrease of 2.0 K to an increase of 1.1 K. Under RCP 6.0, the projected increases are more pronounced, with total heatwave days ranging from 7 to 343 days, duration changes from –18 to 329 days, intensity variations between –1 and 1.6 K, and cumulative intensity ranging from –53 to 871 K x days across the continent. The high-emission scenario (RCP 8.5) projects the most severe impacts, with the maximum change in total heatwave days extending by up to 355 days, duration increasing up to 358 days, intensity changes increasing up to 3.4 K, and cumulative intensity reaching a maximum of 1,721 K x days.

## Discussion

This study provides the first comprehensive evaluation of LSWT dynamics across South America, spanning both historical (1981–2020) and projected future periods (2021–2099). Our results indicate that rising air temperatures and associated changes in surface energy fluxes are driving significant LSWT increases across the continent. Under a high-emission scenario (RCP 8.5), LSWT anomalies could rise by an average of 2.8 ± 0.2 K by the end of the century, with the TH region projected to warm most rapidly (+3.6 ± 0.2 K). These trends are consistent with global projections of lake warming (Grant et al. 2021) and align with regional climate forecasts predicting increases of +3 °C to +5 °C in South American air temperatures (Llopart et al. 2020). Historical observations already show increased warming, more frequent heat extremes, and reduced precipitation, especially in the Amazon and Andes (Seneviratne et al. 2021).

The ecological implications of rising LSWTs are profound. Warmer water temperatures can extend stratification periods, reduce vertical mixing, and accelerate oxygen depletion in deeper layers, leading to hypoxic or anoxic conditions that compromise aquatic life (Wagner and Adrian 2009; Jane et al. 2021). These conditions also favour harmful algal blooms, which degrade water quality and alter species composition, posing major risks for biodiversity, particularly in South America’s highly diverse freshwater ecosystems (Reis et al. 2016; Campos et al. 2021). Our findings also highlight a significant intensification of lake heatwaves across most of South America. By 2099, lake heatwaves are projected to become more frequent, longer, and more intense, especially in the NH, SH, and TH regions. This trend reflects broader climate extremes and mirrors warming patterns observed globally (Woolway et al. 2021a; Wang et al. 2024). These events pose risks not only to aquatic ecosystems but also to water-dependent livelihoods, such as fisheries and aquaculture. The 2023 Amazon heatwave, which caused mass mortality of fish and dolphins and triggered harmful microalgal blooms (Mendes et al. 2024; Fleischmann et al. 2024), underscores the urgency of addressing these threats. Projections suggest northern South America and coastal areas will be particularly vulnerable, demanding adaptive management that considers both ecological sensitivity and social vulnerability (Feron et al. 2019; Ramarao et al. 2024).

A key contribution of this study is the introduction of a diurnal–seasonal thermal typology, which provides a novel lens for comparing lake thermal responses across South America. By classifying lakes based on their DTR, STR, and the DTR/STR ratio, we identified four distinct thermal response types: Thermally Extreme, Thermally Buffered, Seasonally Stable, and Diurnally Volatile. These categories reflect differences in the amplitude and timing of lake thermal variability rather than absolute vulnerability to climate change. Thermally Extreme lakes, characterized by high variability at both diurnal and seasonal scales, were concentrated in southeastern South America, whereas Thermally Buffered lakes, exhibiting low variability, were mainly found in the north. Seasonally Stable lakes, with limited diurnal but high seasonal variation, were primarily located at high altitudes in the Andes. These distinctions are ecologically meaningful: high DTR may increase stress on temperature-sensitive organisms, while high STR may influence phenological cues. Importantly, observed patterns of long-term warming and heatwave exposure do not always align with the typology, indicating that other factors—such as lake depth, basin morphometry, and local meteorology—also play key roles. Thus, while the typology provides a framework to assess relative thermal responsiveness, interpretation of lake vulnerability requires consideration of these additional physical and climatic characteristics. A further limitation concerns the definition of future heatwaves. As noted by Amaya et al. 2023; thresholds based on historical extremes may label conditions at the end of the century as ‘heatwaves,’ even though such temperatures may no longer be anomalous relative to the contemporaneous climate. Consequently, projected future heatwave metrics should be interpreted with caution, as they may conflate persistent warming with truly exceptional events.

Our driver analysis identified air temperature and shortwave radiation as the dominant controls of both LSWT and DTR trends, consistent with previous studies in both tropical and temperate systems (Livingstone and Dokulil 2001; O’Reilly et al. 2015; Schmid and Köster 2016). In particular, the influence of solar radiation was most pronounced in regions with minimal cloud cover, such as the SH region, which includes the Atacama Desert. Conversely, in the NH region near the Equator, the Intertropical Convergence Zone (ITCZ) likely dampens diurnal heating by increasing humidity and cloud cover, leading to observed declines in DTR. In addition to atmospheric drivers, lake-specific factors such as morphometry, elevation, and geographic setting strongly modulate thermal behaviour. High-altitude and deep lakes often buffer against short-term thermal fluctuations, while shallow and exposed lakes respond more acutely to atmospheric forcing (Gunkel and Casallas 2002; Augusto-Silva et al. 2019). Consistent with this, we also find that elevation is highly associated with lake thermal variability, with DTR decreasing and STR increasing along the elevation gradient, resulting in a decline in the DTR/STR ratio at higher elevations (Fig. S12-S14). This relationship is supported by both correlation analyses and group-based comparisons, which show that high DTR/STR lakes are mainly located at lower elevations while low-ratio lakes are primarily at higher elevations. This pattern indicates a transition from diurnally dominated thermal regimes in lowland lakes to seasonally dominated regimes in high-altitude systems, reflecting differences in thermal inertia and climatic forcing. These findings emphasize the value of integrating lake attributes with climate variables to explain LSWT variability. However, it is important to interpret these attribution results with a methodological caveat. While our sensitivity experiments effectively quantify the individual contributions of meteorological drivers to LSWT and DTR trends, this approach inherently decouples variables that are physically correlated in the climate system (e.g., the coupling between solar radiation and air temperature via cloud cover). Consequently, the attribution results should be understood as the isolated theoretical sensitivity of lakes to specific atmospheric forcings, rather than a full representation of the complex, coupled feedback mechanisms occurring in nature.

The scarcity of in-situ temperature measurements in South America represents an important limitation of this study. Although the observed LSWT trends and heatwave patterns are consistent with regional climatic changes, the absence of extensive ground-based validation introduces uncertainty in the precise magnitude of warming and extreme event metrics. This underscores the need for expanded monitoring networks to improve the accuracy of future assessments of lake thermal dynamics across the continent. We also note that precipitation was not explicitly included as a driver in our analysis because it is not an input variable in the FLake model used by GLAST. While rainfall can influence lake surface temperature through multiple mechanisms, including cooling via direct heat flux, convective mixing, and kinetic energy flux, as observed in tropical lakes (Rooney et al. 2018), these effects are generally small relative to the primary atmospheric drivers of LSWT included in this study. The main potential impact of precipitation in lakes may occur indirectly through changes in water clarity, as terrestrial material washed into lakes can alter light attenuation and therefore the penetration of solar radiation. In FLake, the light attenuation coefficient (Kd) is treated as a fixed parameter, and in GLAST this parameter is calibrated for each lake, thereby capturing the non-varying effect of water clarity on lake temperature. Consequently, while precipitation-driven variability in Kd is not accounted for, the model indirectly represents average light penetration effects, and our results should be interpreted with this limitation in mind.

Finally, it is important to consider that our future projections rely on climatic forcing from the CMIP5 ensemble. While CMIP6 represents the latest generation of climate models, CMIP5 was retained in this study to ensure methodological consistency with the established structure of the GLAST dataset. Our analysis comparing the two generations over the overlapping 2015–2099 period reveals that CMIP6 forcing generally projects stronger increasing trends in key meteorological drivers, particularly longwave radiation and air temperature, relative to the CMIP5 ensemble (not shown here). Consequently, the lake warming trends and heatwave intensifications reported here likely represent a conservative estimate compared to projections derived from the latest CMIP6 standards. Future updates to regional lake assessments should aim to incorporate ISIMIP3b (CMIP6) forcing to capture these evolving climate sensitivities.

Our results underscore the urgency of improving lake monitoring systems in South America, particularly given the limited availability of in situ data in many regions. The FLake model and satellite-derived datasets used in this study offer a scalable alternative, but their accuracy, especially for tropical lakes, remains constrained by sparse validation data. Expanding high-frequency temperature monitoring and improving data sharing networks would enhance model calibration and deepen our understanding of lake-climate interactions. Finally, the introduction of a diurnal–seasonal typology provides a valuable framework for future lake research globally. Applying this classification across other continents could help identify generalizable patterns of lake thermal sensitivity and inform adaptive water management in the face of accelerating climate change.

## Conclusions

This study provides the first continent-wide assessment of historical and projected LSWT dynamics across South America, incorporating both diurnal and seasonal thermal patterns from 1981 to 2099. We found that 97.0% of lakes exhibited a significant long-term warming trend, averaging +0.11 K decade^-1^, while 86.2% showed increasing diurnal variability (+0.02 K decade^-1^) over the past four decades. Air temperature and solar radiation emerged as the dominant drivers of these trends, with solar radiation particularly influencing short-term (diurnal) fluctuations. Projections under different greenhouse gas scenarios indicate that LSWTs will continue to rise across all thermal regions, with warming rates ranging from +0.02–0.03 K decade^-1^ under RCP 2.6 to +0.3–0.4 K decade^-1^ under RCP 8.5 by the end of the 21st century. Correspondingly, lake heatwaves are expected to become significantly more severe. Under the high-emission scenario, heatwave durations could extend by up to 358 days and intensities may increase by as much as 3.4 K. Importantly, this study introduces a new typology of lake thermal responses based on diurnal and seasonal temperature ranges (DTR and STR). This framework reveals distinct lake types, such as Thermally Extreme, Thermally Buffered, Seasonally Stable, and Diurnally Volatile, which reflect different sensitivities to atmospheric forcing. These classifications offer a valuable tool for identifying lakes most vulnerable to climate change and for prioritizing future monitoring and management efforts. Altogether, this work enhances our understanding of how South American lakes are responding to a changing climate and underscores the need for targeted, region-specific adaptation strategies. Continued integration of high-resolution modelling, satellite data, and improved in situ observations will be critical for safeguarding freshwater ecosystems and resources in the decades ahead.

## Supplementary Information

Below is the link to the electronic supplementary material.

Supplementary Material 1

## Data Availability

The GLAST dataset is available at https://zenodo.org/records/8322038.
