# Supplementary material for: Emerging changes in lake temperature extremes and variability in South America
Source: Clim Change. 2026 Feb 16;179(3):41. doi: 10.1007/s10584-026-04137-0 (PMC12909474; doi:10.1007/s10584-026-04137-0)
Supplement: Supplementary file 1 — Supplementary Material 1 [file 10584_2026_4137_MOESM1_ESM.docx]

**Title:** Emerging changes in lake temperature extremes and variability in South America

**Supplementary Information**


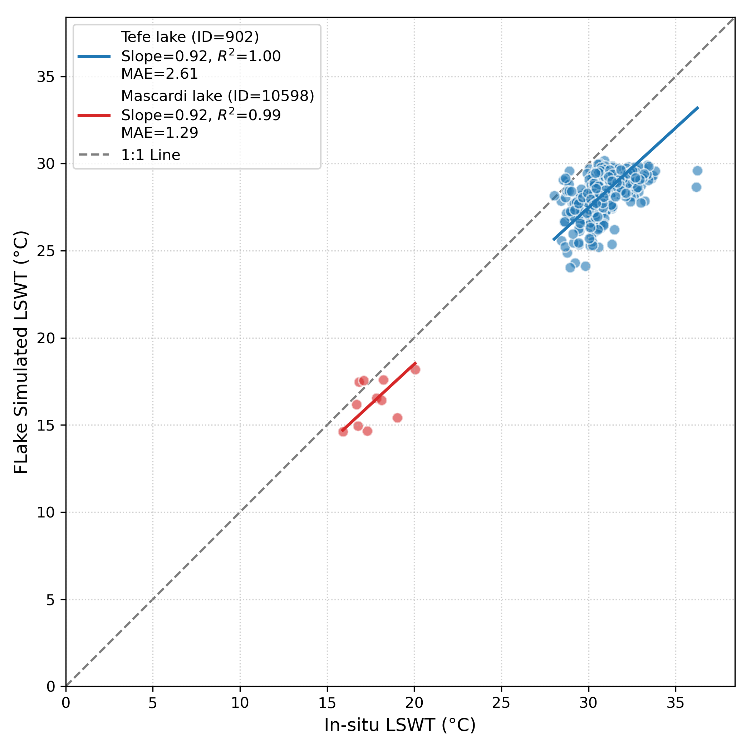


**Figure S1.** Comparison of simulated versus observed daily LSWT for Tefe Lake (HydroID 902) and Mascardi Lake (HydroID 10598). Solid lines denote the zero-intercept linear regression models, and the dashed line represents the 1:1 line. Model performance metrics (Slope, R^2^, and MAE) are shown in the legend.


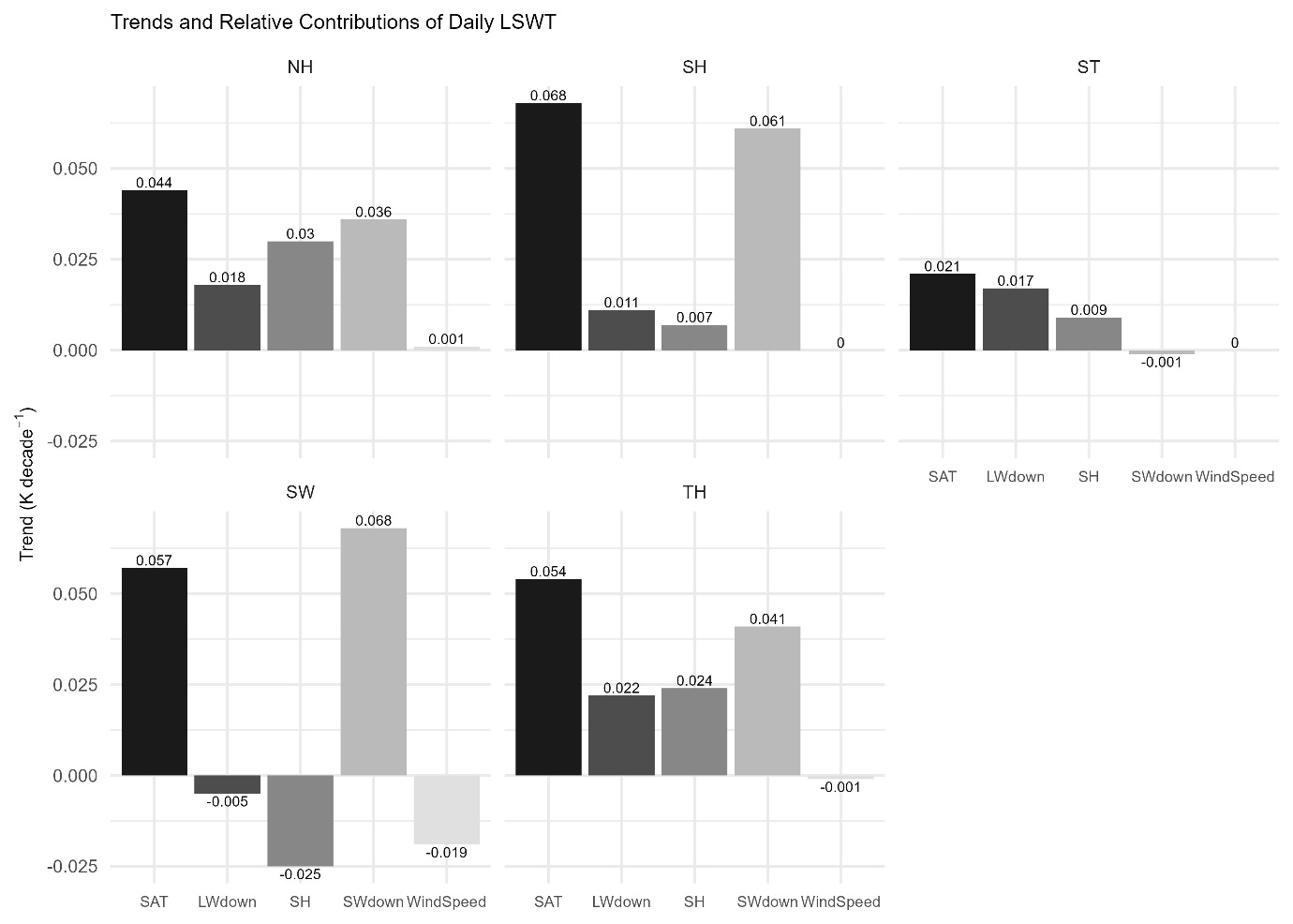


**Figure S2.** Trend and relative contribution of daily LSWT (SAT- Surface Air Temperature, LWdown- Longwave Downward Radiation, SH- Specific Humidity, SWdown- Shortwave Downward Radiation, WindSpeed- Wind Speed) per thermal regions (NH- Northern Hot, SH- Southern Hot, ST- Southern Temperate, SW- Southern Warm, TH- Tropical Hot).


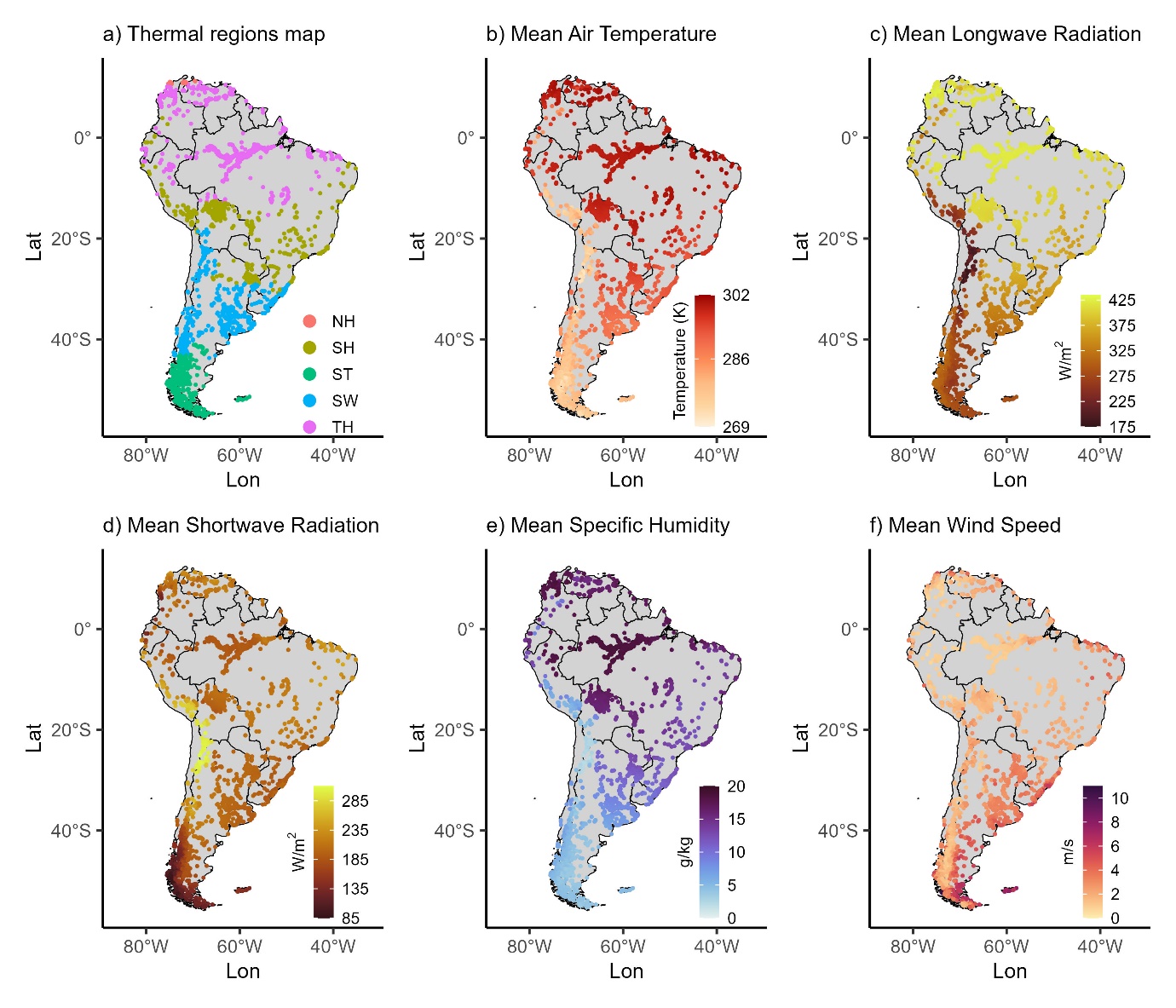


**Figure S3.** Mean daily meteorological variables from 1981 to 2020- (b) air temperature, (c) longwave downward radiation, (d) shortwave downward radiation, (e) specific humidity, (f) wind speed.


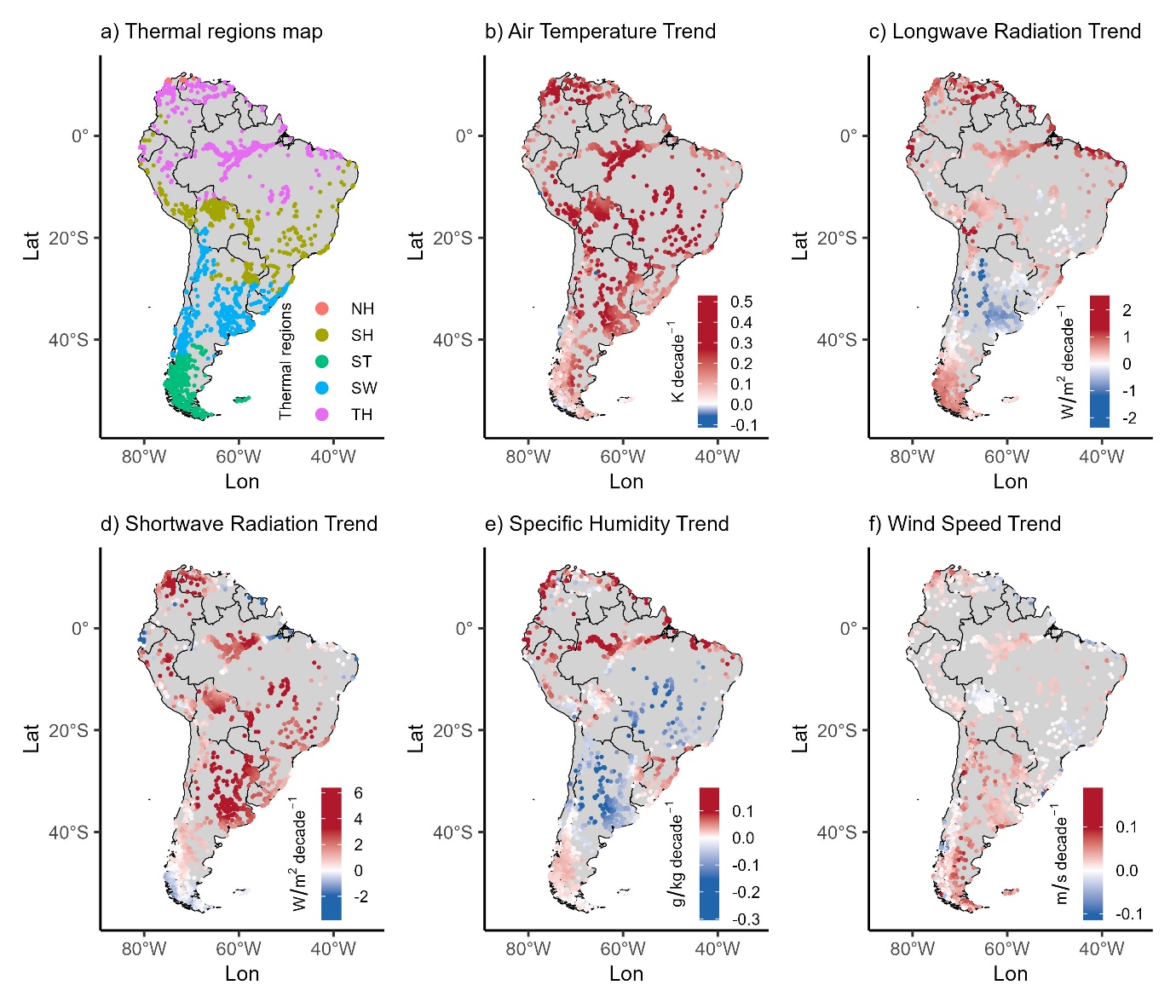


**Figure S4.** Trends in daily meteorological variables from 1981 to 2020- (a) Maps of thermal regions in South America (NH- Northern Hot, SH- Southern Hot, ST- Southern Temperate, SW- Southern Warm, TH- Tropical Hot); (b) air temperature, (c) longwave downward radiation, (d) shortwave downward radiation, (e) specific humidity, (f)-wind speed.


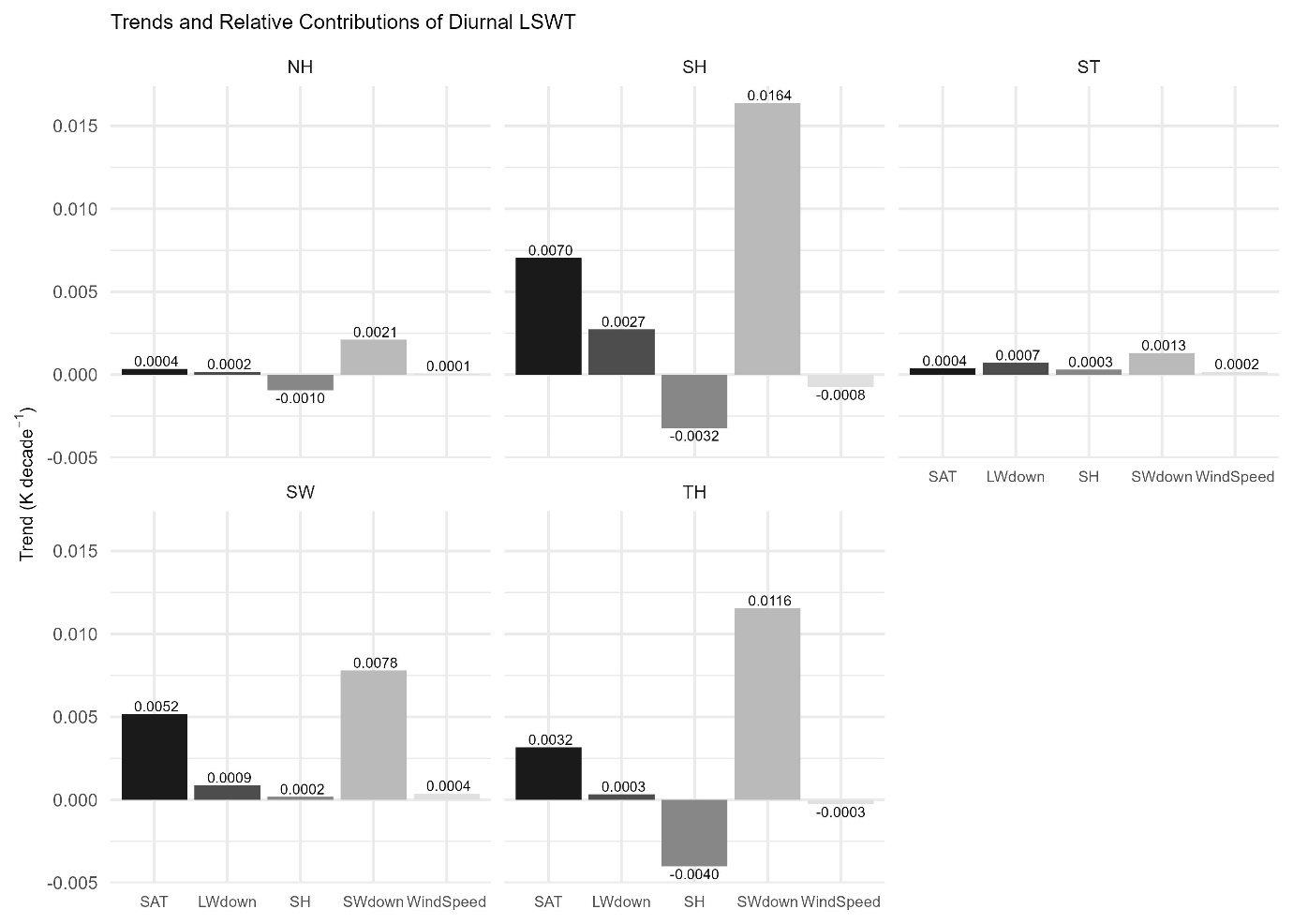


**Figure S5.** Trend and relative contribution of diurnal LSWT per thermal regions (SAT- Surface Air Temperature, LWdown- Longwave Downward Radiation, SH- Specific Humidity, SWdown- Shortwave Downward Radiation, WindSpeed- Wind Speed).

**
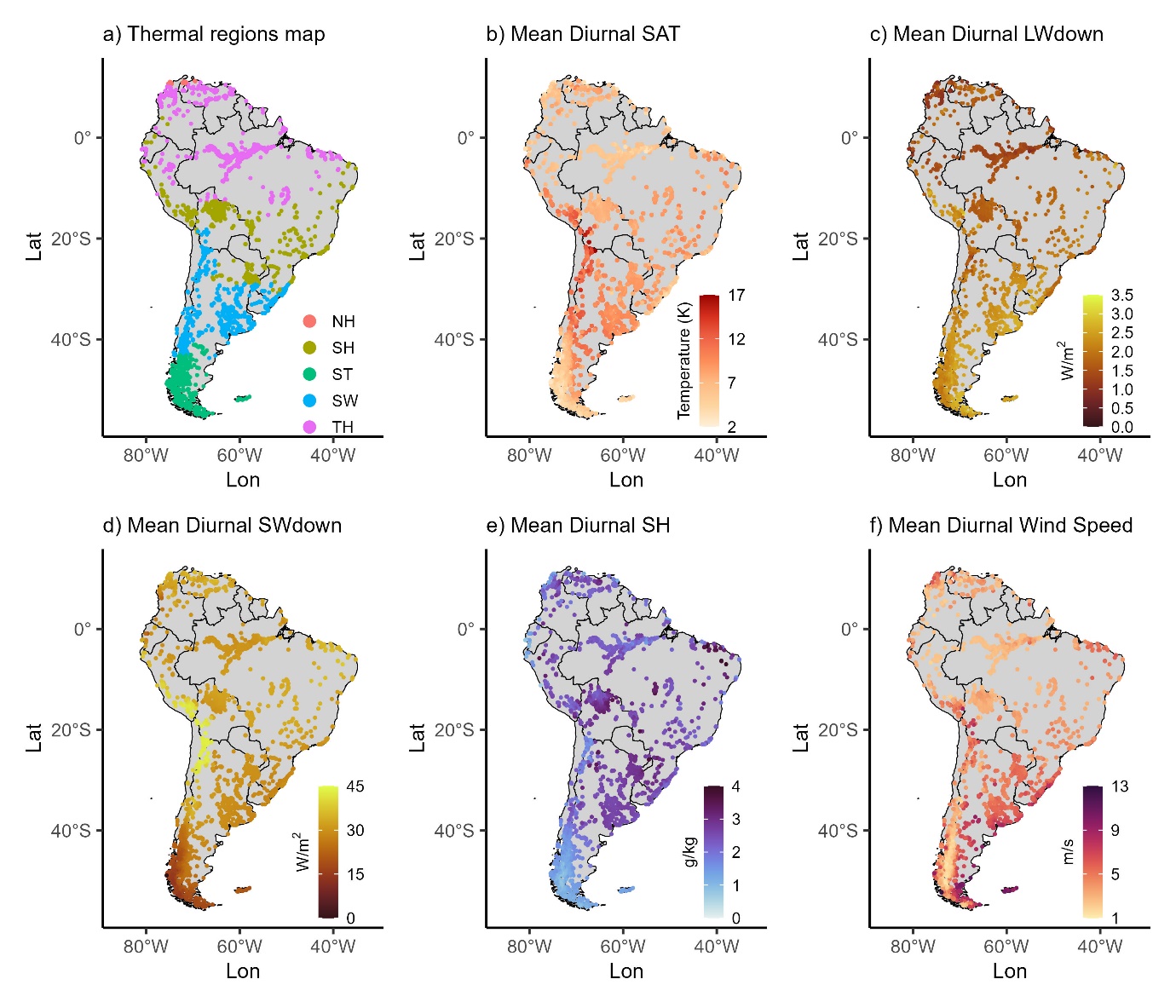
** **Figure S6.** Mean diurnal meteorological variables from 1981 to 2020- (b) air temperature, (c) longwave downward radiation, (d) shortwave downward radiation, (e) specific humidity, (f)-wind speed.


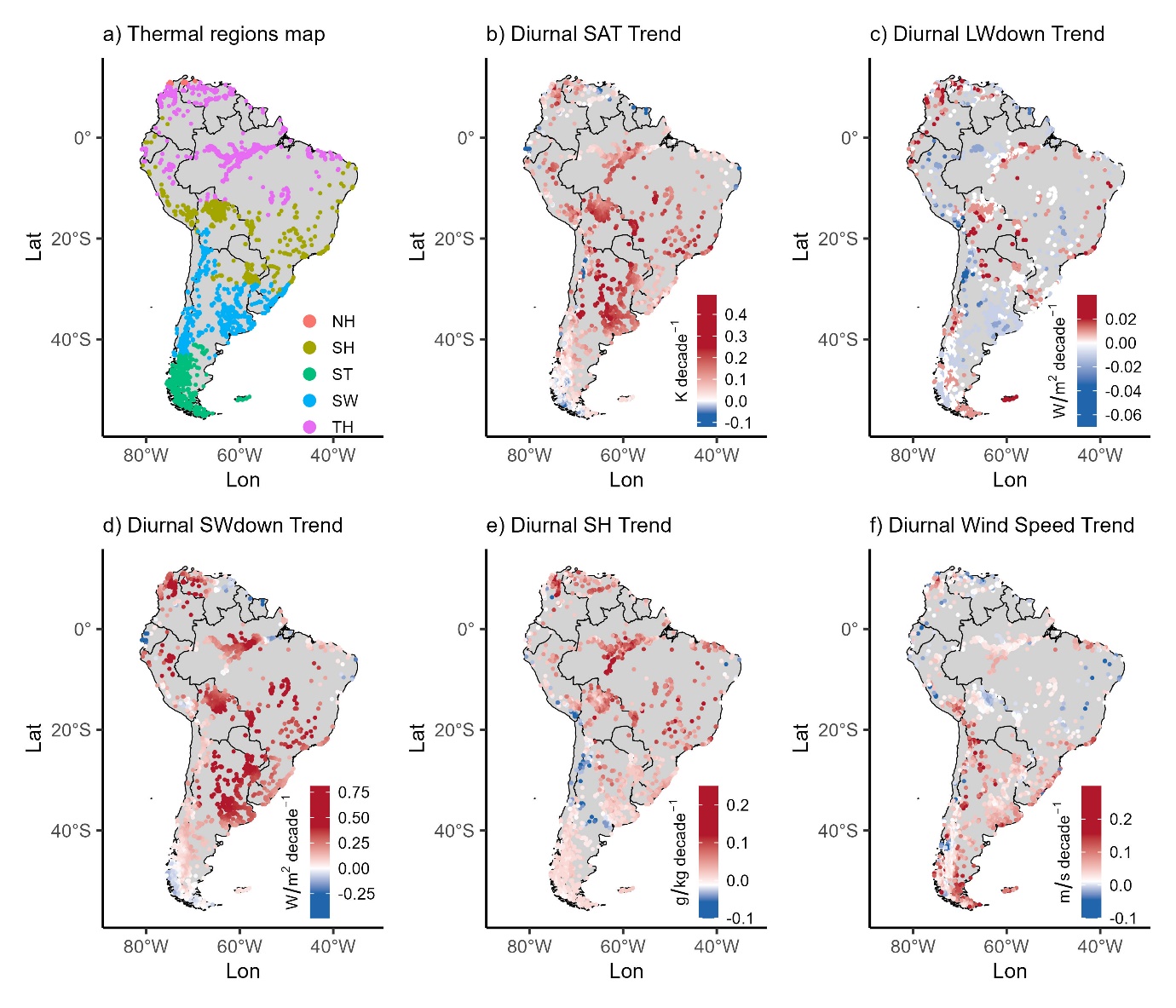


**Figure S7.** Trends in diurnal meteorological variables from 1981 to 2020- (b) surface air temperature, (c) longwave downward radiation, (d) shortwave downward radiation, (e) specific humidity, (f)-wind speed.


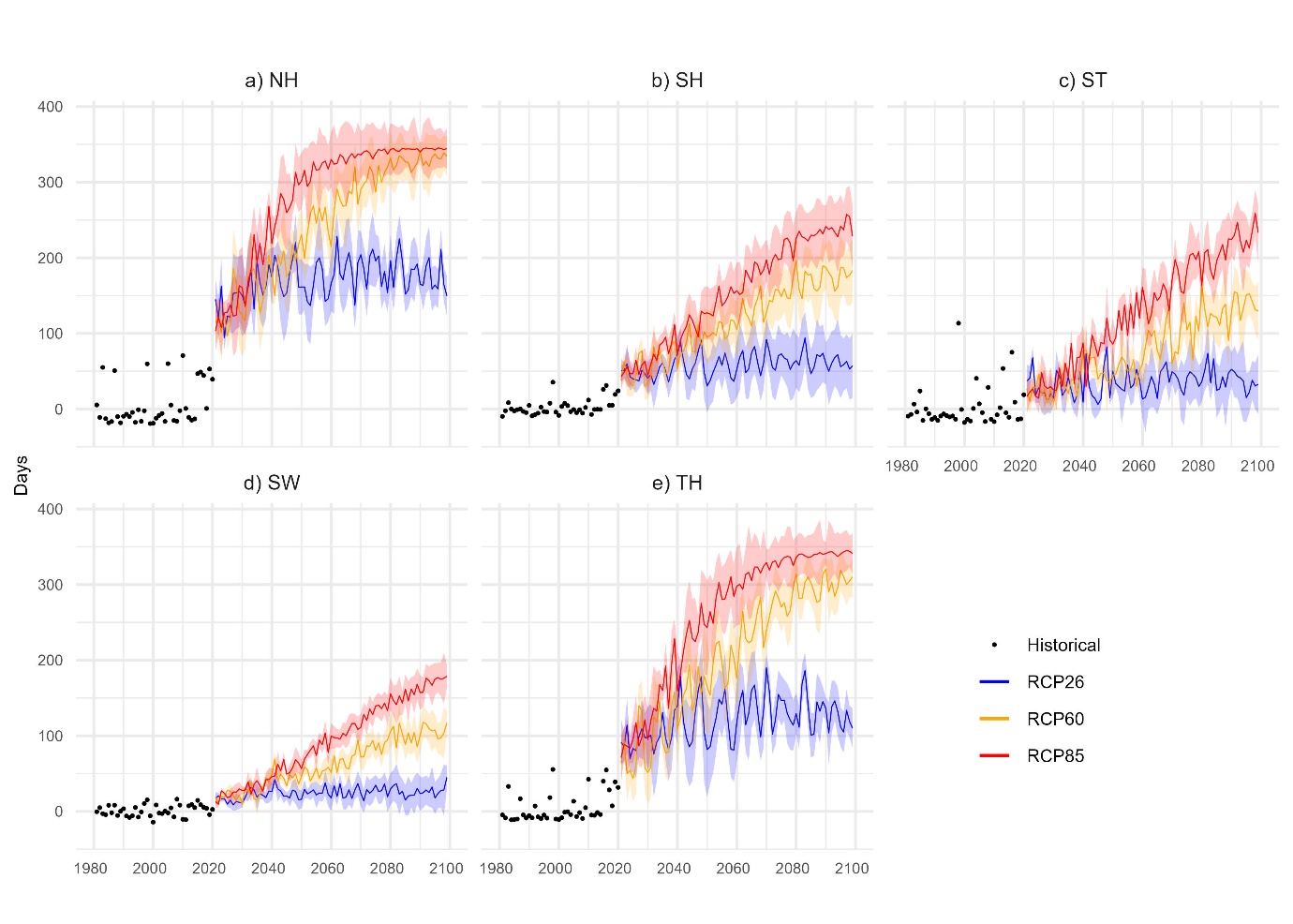


**Figure S8.** Total heatwave day anomalies per thermal regions (a) Northern Hot, (b) Southern Hot, (c) Southern Temperate, (d) Southern Warm, and (e) Tropical Hot. The thick lines represent total heatwave day anomalies and the shaded areas show the standard deviation across the multi-model ensemble. The historical temperature anomalies (black) from 1981 to 2020, the projections under RCP 2.6 (blue), RCP 6.0 (orange), RCP 8.5 (red) scenarios from 2021 to 2099


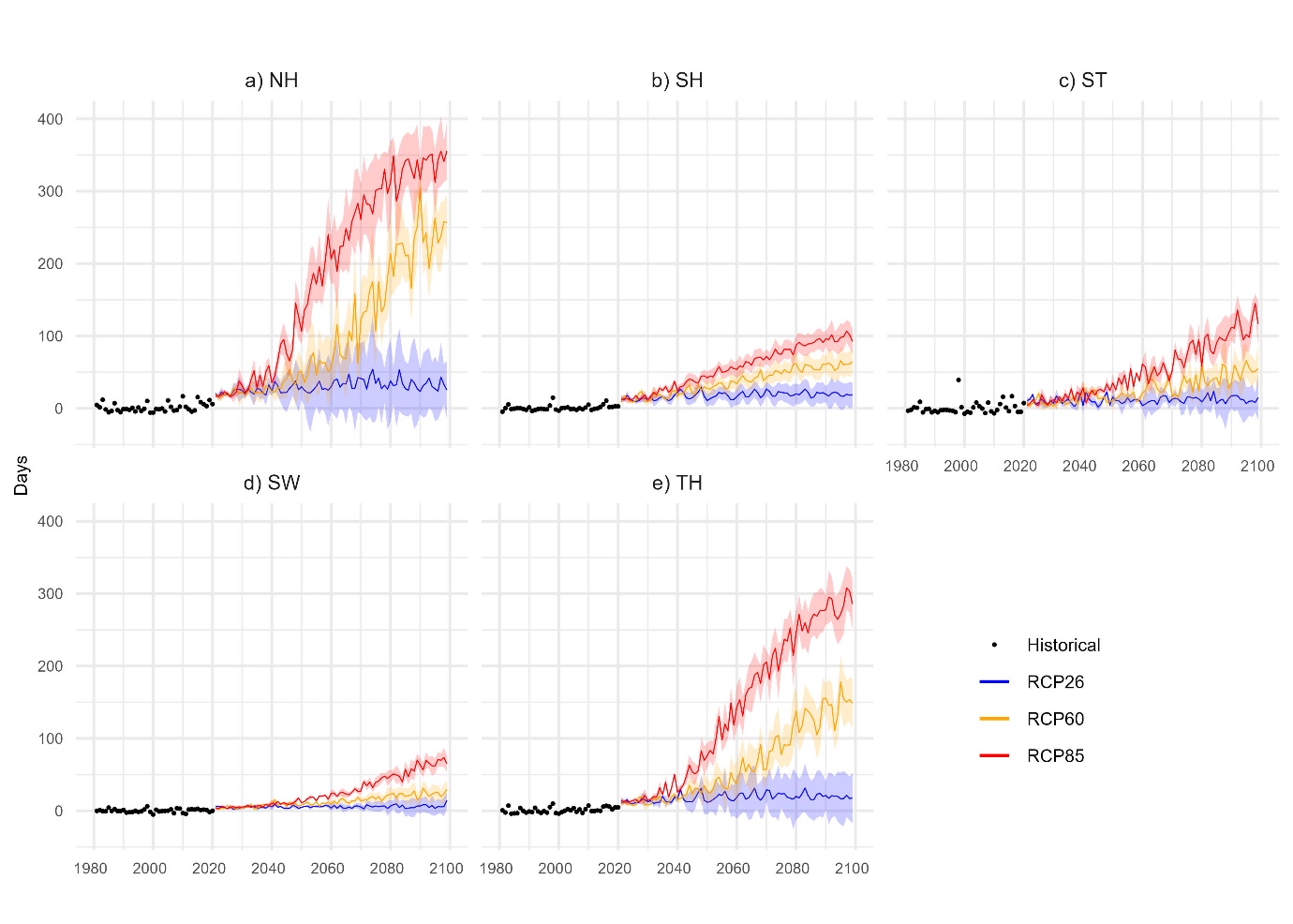


**Figure S9.** Heatwave duration anomalies per thermal regions (a) Northern Hot, (b) Southern Hot, (c) Southern Temperate, (d) Southern Warm, and (e) Tropical Hot. The thick lines represent average anomalies and the shaded areas show the standard deviation across the multi-model ensemble. The historical temperature anomalies (black) from 1981 to 2020, the projections under RCP 2.6 (blue), RCP 6.0 (orange), RCP 8.5 (red) scenarios from 2021 to 2099.


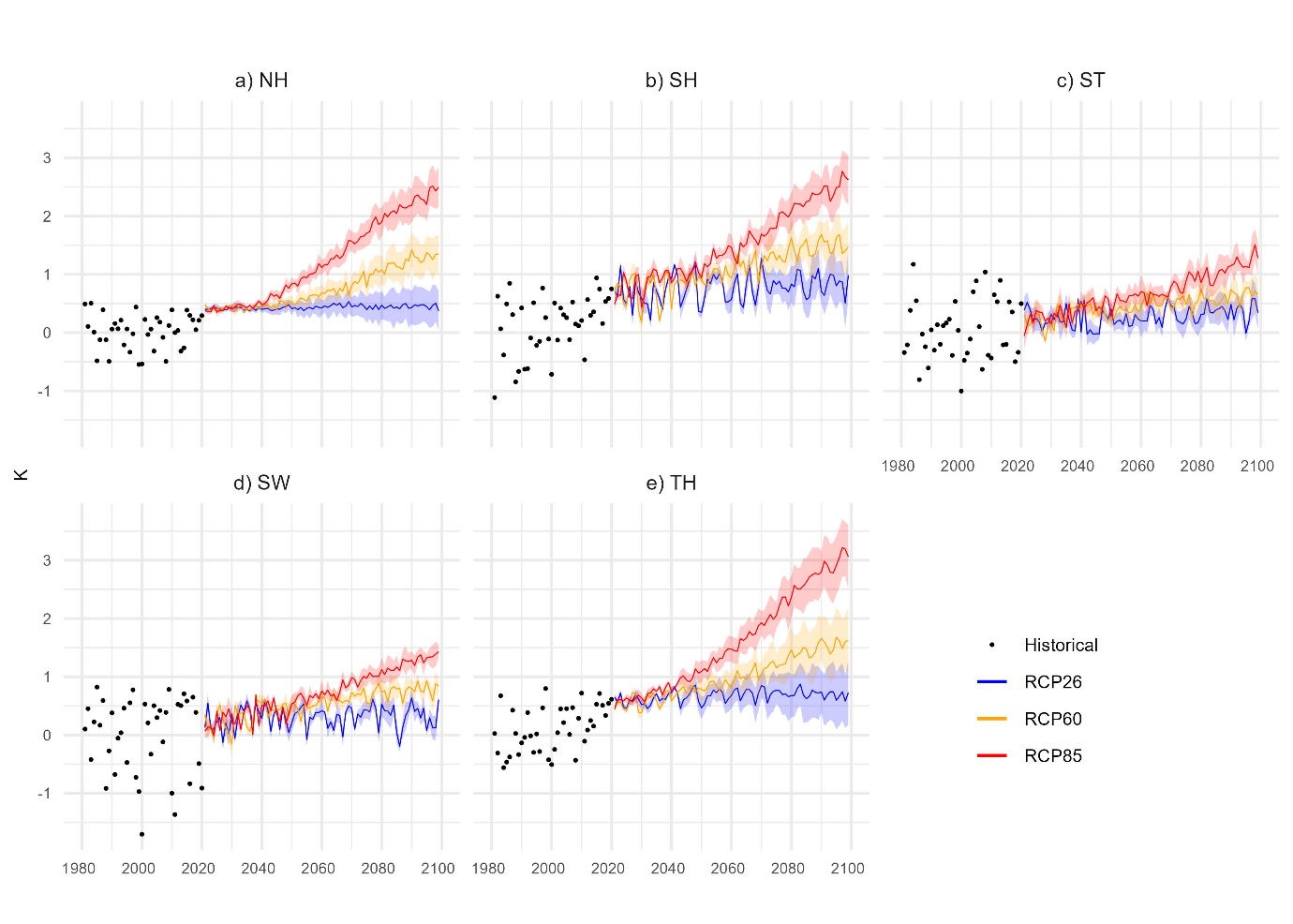


**Figure S10.** Heatwave intensity anomalies per thermal regions (a) Northern Hot, (b) Southern Hot, (c) Southern Temperate, (d) Southern Warm, and (e) Tropical Hot. The thick lines represent average anomalies and the shaded areas show the standard deviation across the multi-model ensemble. The historical temperature anomalies (black) from 1981 to 2020, the projections under RCP 2.6 (blue), RCP 6.0 (orange), RCP 8.5 (red) scenarios from 2021 to 2099.


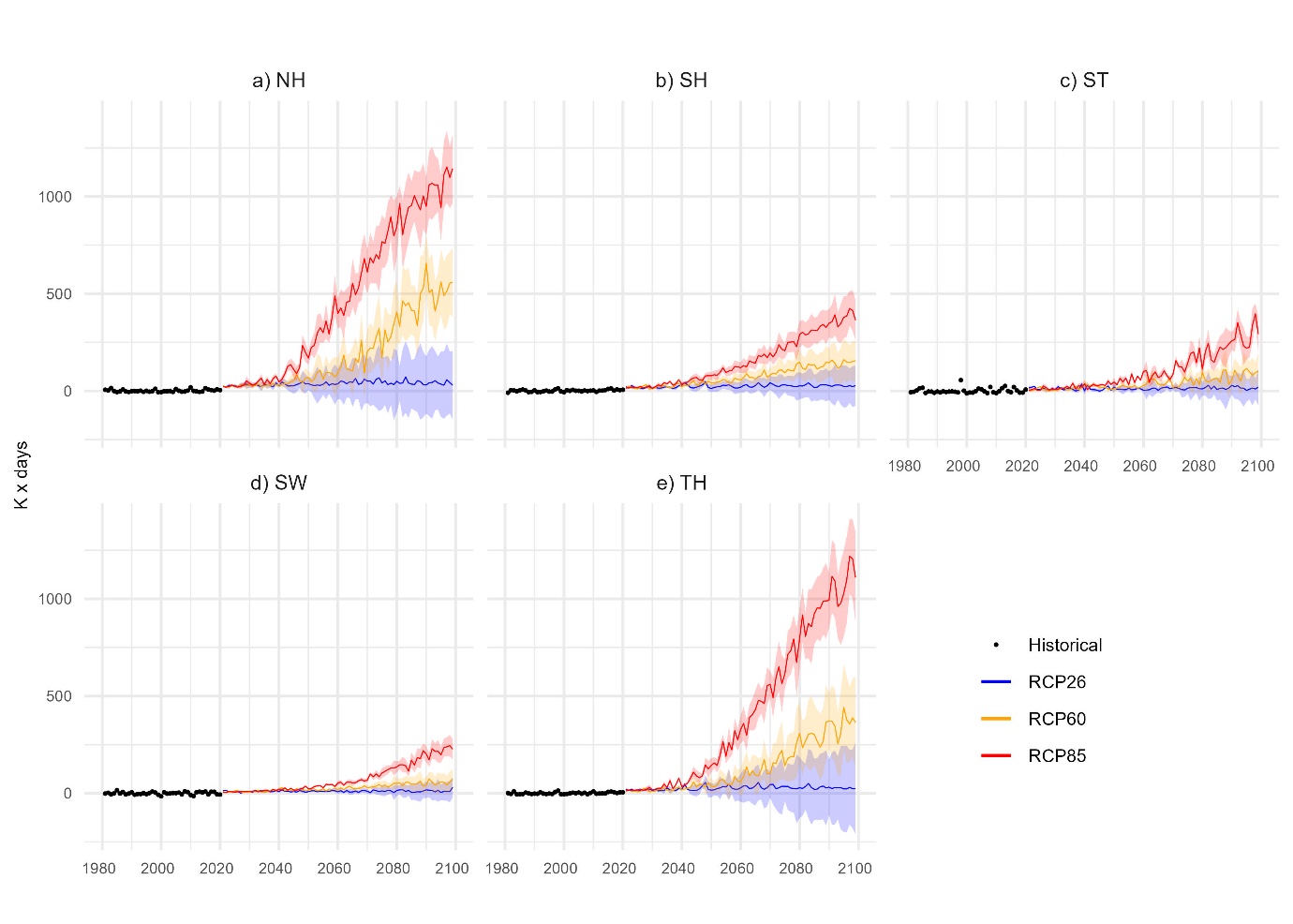


**Figure S11.** Heatwave cumulative intensity anomalies per thermal regions (a) Northern Hot, (b) Southern Hot, (c) Southern Temperate, (d) Southern Warm, and (e) Tropical Hot. The thick lines represent average anomalies and the shaded areas show the standard deviation across the multi-model ensemble. The historical temperature anomalies (black) from 1981 to 2020, the projections under RCP 2.6 (blue), RCP 6.0 (orange), RCP 8.5 (red) scenarios from 2021 to 2099.


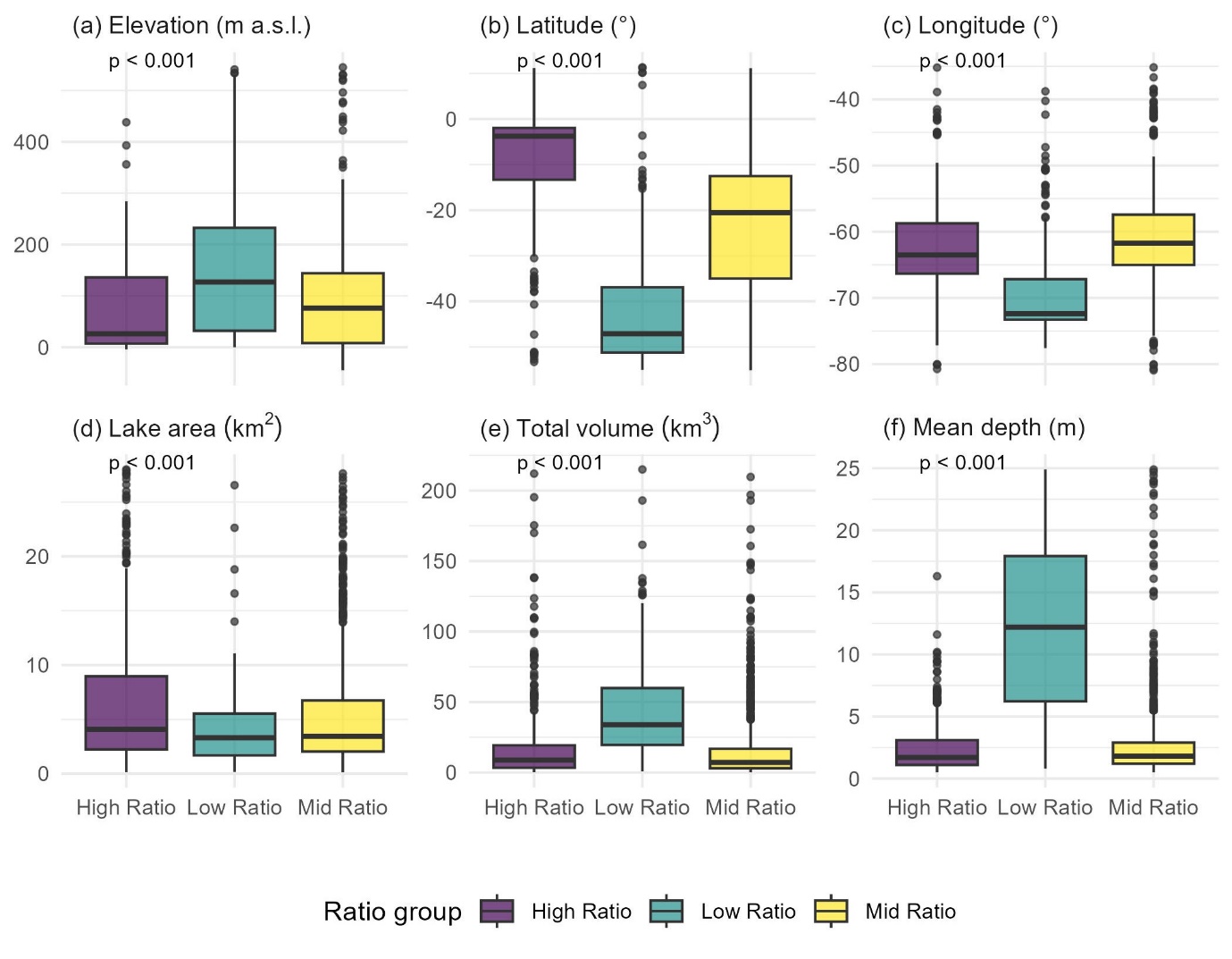


**Figure S12.** Comparison of geographic and morphometric drivers of lake thermal ratios across Low, Mid and High DTR/STR ratio groups.


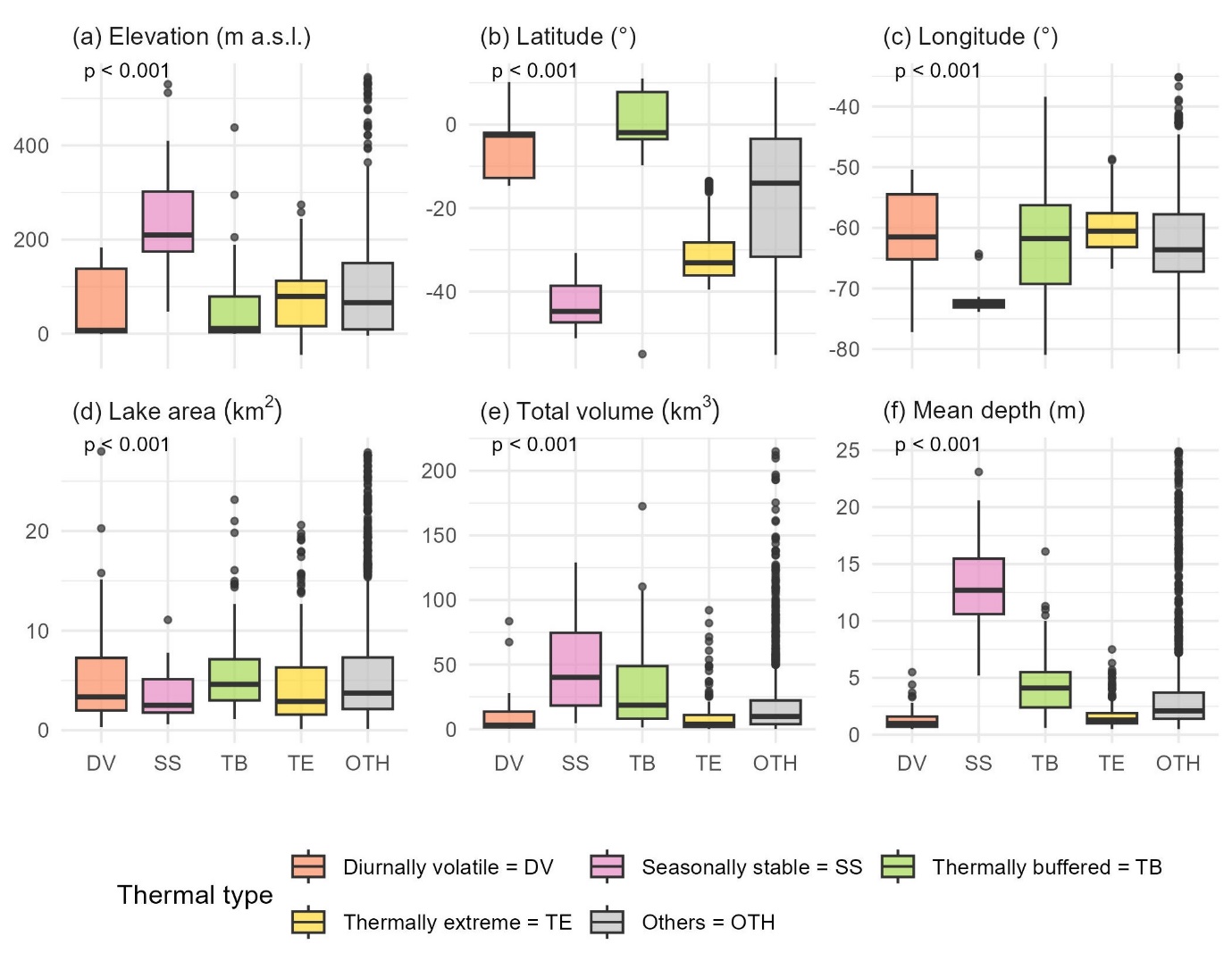


**Figure S13.** Comparison of geographic and morphometric drivers across thermal response types.


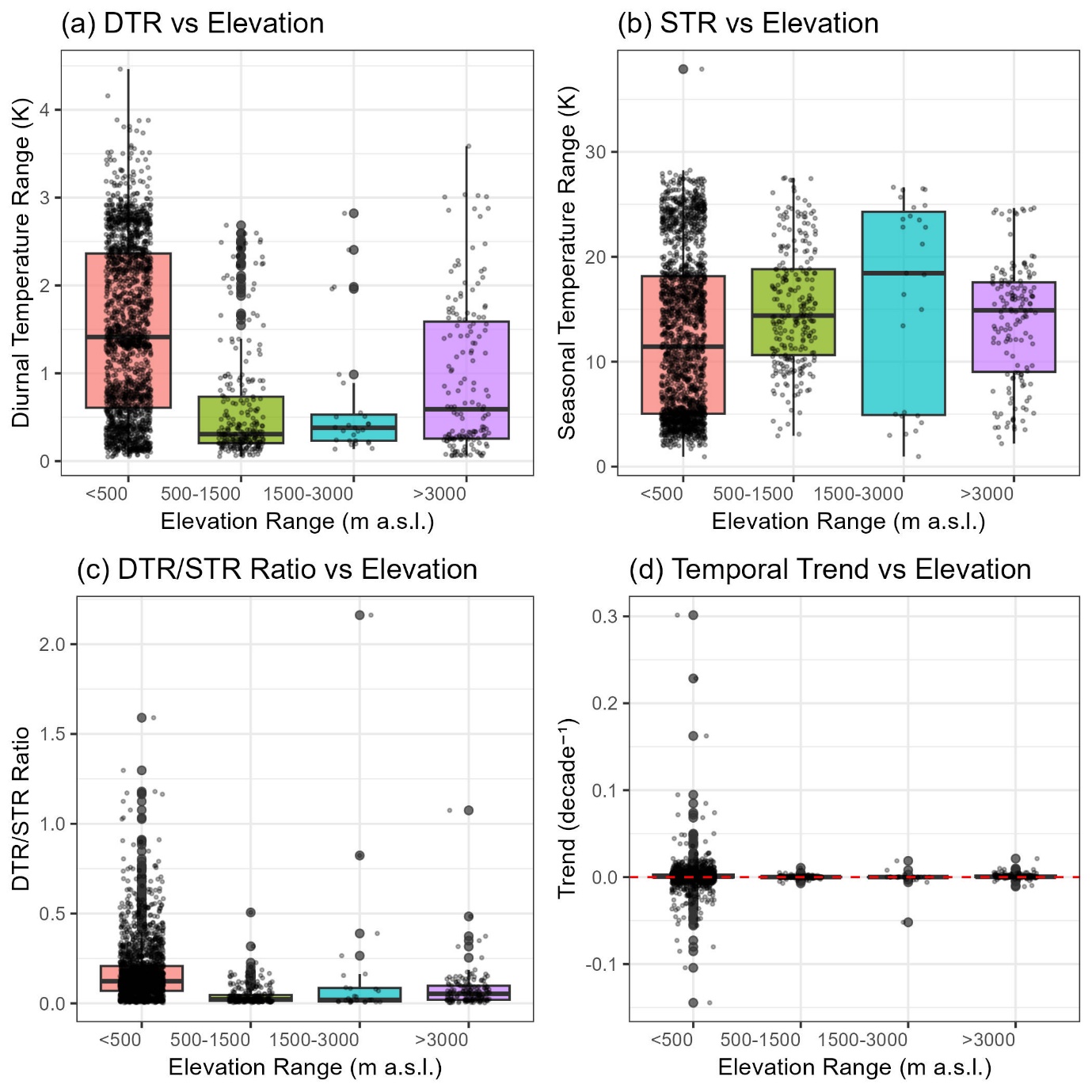


**Figure S14.** Elevation range-driven shifts in diurnal and seasonal lake thermal variability.
